# Utilizing Surface Acoustic Wave Nebulization (SAWN) for the Rapid and Sensitive Ambient Ionization Mass Spectrometric Analysis of Organic Explosives

**DOI:** 10.1007/s13361-019-02335-y

**Published:** 2019-10-28

**Authors:** Lauren Pintabona, Alina Astefanei, Garry L. Corthals, Arian C. van Asten

**Affiliations:** 1 van ‘t Hoff Institute for Molecular Sciences, Faculty of Science, University of Amsterdam, PO Box 94157, 1090 GD Amsterdam, The Netherlands; 2 CLHC, Amsterdam Center for Forensic Science and Medicine, University of Amsterdam, P.O. Box 94157, 1090 GD Amsterdam, The Netherlands

**Keywords:** SAWN, Surface acoustic wave nebulization, Ambient ionization mass spectrometry, Explosives, Forensic science, Security

## Abstract

When considering incident investigations and security checks focused on energetic materials, there is an ongoing need for rapid, on-scene chemical identification. Currently applied methods are not capable of meeting all requirements, and hence, portable mass spectrometry is an interesting alternative although many instrumental challenges still exist. To be able to analyze explosives with mass spectrometry outside the traditional laboratory, suitable ambient ionization methods need to be developed. Ideally such methods are also easily implemented in the field requiring limited to no power sources, gas supplies, flow controllers, and heating devices. For this reason, the potential of SAWN (surface acoustic wave nebulization) for the ambient ionization and subsequent mass spectrometric (MS) analysis of organic explosives was investigated in this study. Excellent sensitivity was observed for nitrate-based organic explosives when operating the MS in negative mode. No dominant adduct peaks were observed for the peroxides TATP and HMTD with SAWN-MS in positive mode. The MS spectra indicate extensive fragmentation of the peroxide explosives even under the mild ionization conditions provided by SAWN. The potential of SAWN-MS was demonstrated with the correct identification of nitrate-based organic explosives in pre- and post-explosion case samples in only a fraction of the time and effort required for the regular laboratory analysis. Results show that SAWN-MS can convincingly identify intact organic energetic compounds and mixtures but that sensitivity is not always sufficient to detect traces of explosives in post-explosion residues.

**Electronic supplementary material:**

The online version of this article (10.1007/s13361-019-02335-y) contains supplementary material, which is available to authorized users.

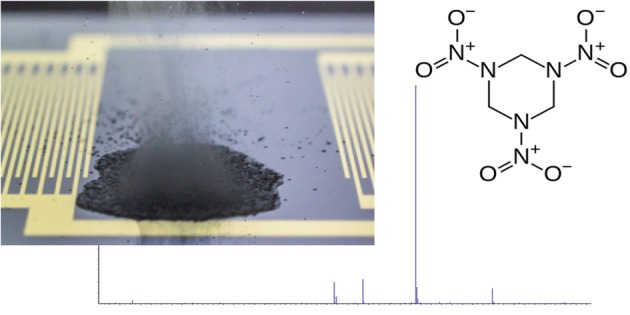

## Introduction

When law enforcement and forensic professionals must deal with a potential incident involving energetic materials, or when security personnel need to check items and persons for the presence of explosives, there is an ongoing need for rapid chemical analysis-based screening and detection methods that can be employed in the field. Given the high risk involved, such methods should exhibit superior sensitivity and selectivity. If methods are not sensitive enough, false-negative results could lead to explosive material not being detected with potentially dramatic consequences. On the other hand, insufficient selectivity may lead to too many false-positive outcomes, which could cause disruption, delays, and false accusations. In addition to these stringent demands, fieldable methods should be able to cover multiple explosives of interest in a single analysis, should be as non-invasive as possible (to prevent sampling activated explosions), should be very fast (especially in a security setting with mass screening), should be easy to use (preferably by professionals with limited knowledge on the chemistry and chemical analysis of explosives), should be extremely robust (ensuring minimal down-time of the equipment and quality of the results), and should be affordable (thereby limiting the on-cost of the investigation and screening).

Methods that currently are used in an actual operational forensic and security setting to detect and characterize traces of explosives include colorimetric assays, portable IR and Raman spectroscopy, and ion mobility spectrometry. These methods have proven to be sufficiently robust to be used by nonexperts outside the controlled environment of the laboratory. To this end, commercial companies provide tailor-made equipment, methods, consumables, and on-site support. Although new scientific and technological developments are reported continuously [1–5], it can be argued that none of these techniques currently meet all the requirements as formulated above. Especially limited sensitivity and selectivity can raise a concern for false negative and false positive outcomes that can have very serious consequences when it comes to on-scene explosives analysis [6].

As the search for the ideal method for the field analysis of explosives thus continues, many scientists have advocated the use of mass spectrometry (MS) with ambient ionization for trace explosive analysis. Indeed, from a theoretical point of view, mass spectrometry seems capable of fulfilling many of the listed requirements. The technique is sensitive, selective (especially when considering high resolution, MS/MS, and/or MS^n^ modes of operation), versatile (tailor made methods can be developed on a single instrument to screen for a wide array of compounds of interest), and often offers near real-time analysis. Unfortunately, this theoretical potential has so far been impeded by the huge technological challenges that exist when considering fieldable mass spectrometry. MS requires powerful pumps to maintain the high vacuum conditions needed for the analysis, especially when considering ionization of the analytes of interest under ambient conditions. Such pumps are difficult to miniaturize and typically have high power consumption. However, promising technical progress has been reported for point-of-care and even on-scene, portable MS systems [7, 8].

In anticipation of technological breakthroughs to realize fieldable MS systems, the scientific community has studied and developed methods for the ambient ionization of explosives in the laboratory with high end MS equipment. This revealed that the ionization of explosives represents a challenge in itself. Nitro-based organic energetic materials are typically analyzed in negative mode and often require the addition of dopants to effectively ionize through the formation of adduct ions. In contrast, peroxide-based explosives like TATP (triacetone triperoxide) and HMTD (hexamethylene triperoxide diamine) are usually analyzed in positive mode and easily fragment during ionization. The MS analysis of the small inorganic ions associated with pyrotechnic mixtures and inorganic nitrate and chlorate species requires high desorption temperatures and de-clustering at the inlet of the mass spectrometer through in-source CID (collision-induced dissociation) [9]. This makes ambient ionization and subsequent MS analysis of explosives much more challenging than for instance drugs of abuse for which most compounds can easily be ionized by protonation of a Lewis base within their chemical structure.

Of the various ambient ionization techniques DART (direct analysis in real time) has been used for the analysis of both inorganic [9, 10] and organic explosives [11–14] with excellent sensitivity. The design of the DART source also facilitates real-time and high-throughput analysis and seems sufficiently robust and reproducible although ionization can be affected by external factors like humidity [15]. With the introduction of DESI (desorption electrospray ionization) in 2004, the inventors demonstrated the potential of the technique to desorb and analyze explosive traces from surfaces [16]. In follow-up studies, it was shown that DESI enabled the direct analysis of explosives traces on human skin and fabric without any sample pretreatment [17, 18]. The sensitivity of TATP analysis was improved by doping the spray solvent with suitable cations for adduct ion creation [19]. More recently, explosive trace analysis with paper spray ionization mass spectrometry (PS-MS) has been demonstrated [20, 21]. When designed properly, the paper substrate can be used to swipe surfaces but swabs can also be used directly in a similar fashion [22]. Through a reactive paper spray approach involving on-paper catalytic reduction, very low detection limits for TNT (trinitrotoluene) have been reported even enabling vapor detection of this nitroaromate explosive [23].

In addition, several other ambient ionization approaches have been used to effectively and rapidly analyze explosives, including plasma-based techniques [24–28], laser desorption [29–31], and thermal desorption [32, 33] in combination with suitable ionization methods, Corona [34] or dielectric barrier discharge [35, 36] based ionization, electrospray (ESI) and DESI variants [37–40] and photoionization [41].

Although excellent results have been reported, not every ambient ionization technique is easily applied in the field in combination with a portable or point-of-care MS. The need for relatively high gas flows and high temperatures in combination with a substantial power consumption can hamper the use of an ambient ionization method outside the laboratory. In that respect, surface acoustic wave nebulization (SAWN) as introduced in 2010 by Heron et al. [42] could be an interesting alternative. With SAWN a small volume of liquid is placed on a piezoelectric material (typically LiNbO_3_) and effectively nebulized by a surface acoustic wave generated by applying a MHz frequency voltage wave to an interdigitated transducer (IDT) [43]. With a power consumption of only a few watts, the nebulization results in the formation of small droplets that, when introduced into a mass spectrometer, subsequently evaporate and facilitate analyte ionization. It has been shown that ions produced with SAWN possess a lower internal energy compared to electrospray ionization (ESI) [44], making SAWN an interesting technique for the MS analysis of peptides and other high molecular mass biological compounds [45–50]. However, recently, the SAWN-MS analysis of drugs [51], foodstuffs [52], and dyes [53] has also been reported.

To our knowledge, this study is the first to report the successful use of SAWN-MS to analyze organic explosives for forensic and security applications. Despite the fact that SAWN is considered to be a low-energy ionization technique, excellent sensitivity was obtained for nitroorganic explosives using a piezoelectric chip with a dual IDT [54]. This finding creates interesting opportunities for the analysis of energetic materials in the field by coupling a SAWN chip to a portable mass spectrometer.

## Materials and Methods

### Chemicals, Standards, and Case Work Samples

All explosive standards were provided by the Netherlands Forensic Institute (NFI; The Hague, Netherlands) and were solutions of 0.1 or 1 mg/mL, purchased from AccuStandard, Inc. (New Haven, CT, USA). Among these standards were pentaerythritol tetranitrate (PETN), erythritol tetranitrate (ETN), triacetone triperoxide (TATP), hexamethylene triperoxide diamine (HMTD), 2,4,6-trinitrotoluene (TNT), cyclotrimethylenetrinitramine (RDX), 1,3,5,7-tetranitro-1,3,5,7-tetrazocane (HMX), nitroglycerin (NG), and 2,4,6-trinitrophenylmethylnitramine (tetryl). Anonymous case work extracts, both pre- and post-explosion, were also provided by the explosives team of the NFI. The case samples containing traces of explosives in methanol (MeOH) were analyzed as 10-fold dilutions in MeOH. In case additional sensitivity was required, reduced dilution factors and additives were used as specified in the “Results and Discussion” section.

LC-MS grade water, MeOH, and acetonitrile (ACN) were purchased from Biosolve BV (Valkenswaard, Netherlands). HPLC grade, 99.9% isopropanol (IPA), was acquired from Sigma-Aldrich (Darmstadt, Germany). Ammonium acetate (~ 98%), formic acid (< 96%), chloroform, and benzoic acid (> 99.5%) were also purchased from Sigma-Aldrich. Ammonium hydroxide (32%, extra pure) was obtained from Merck (Darmstadt, Germany). TechniSolv, pure ethanol (EtOH), 96%, originated from VWR (Radnor, PA, USA).

### Sample Preparation

Stock solutions (1 μg/mL) of the explosives were diluted with MeOH prior to analysis, with the exception of PETN, which required dilution in MeOH/H_2_O (70/30 *v*/*v*%) with 0.1 *v*% CHCl_3_ additive, and tetryl, which was diluted in MeOH/H_2_O (70/30 *v*/*v*%). The peroxide explosives were prepared in IPA, with 15 *v*% of an aqueous ammonia solution (32 wt%) for TATP, and 15 *v*% of an aqueous formic acid solution (0.1 wt%) for HMTD. Aqueous solutions were prepared and used as such (the pH was not adjusted).

### SAWN Parameters

**Figure 1:**
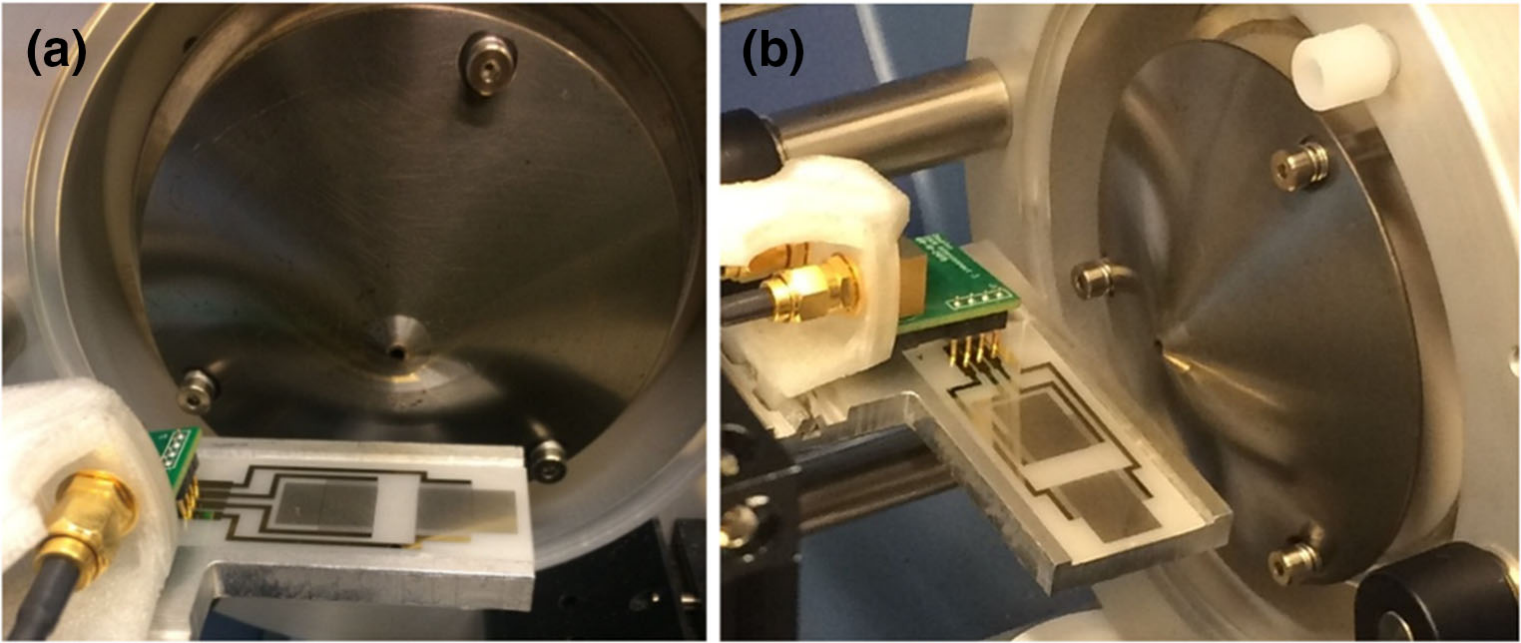
SAWN-MS setup with front view showing chip position, with delay zone centered under the exposed inlet of the TripleTOF 5600+ mass spectrometer (**a**) and side view showing the chip holder with pins (arrow) to provide power to the IDTs (**b**)

The SAWN setup used for this study is described in more detail elsewhere [53]. In short, a small amount of liquid sample is continuously deposited on a piezoelectric chip (LiNbO_3_), etched with interdigitated transducers (IDTs) on either side. The application of power to the IDTs generates acoustic waves (SAWs) that travel along the surface of the substrate and disrupt the liquid sample. This results in a plume of aerosols containing analyte ions, which then directly enter the vacuum interface of the MS, where desolvation and analysis take place in real time. In the current work, the ESI source was removed from the front of the mass spectrometer and replaced with the SAWN chip, which was positioned 1–2 cm below the orifice and centered on the inlet. This experimental setup is depicted in Figure 1. The liquid sample was deposited on the delay zone of the substrate using a micropipette. The SAWN device is operated by an Android tablet connected to an electronic box. Custom-made software on the tablet allows the user to control the power and signal frequency applied to the chip. For each analysis, 6.5 W of power was applied in continuous mode to the IDTs on the SAWN chip. Approximately 2 μL of sample was slowly deposited onto the surface of the chip and quickly nebulized into a plume of aerosols following the application of a signal frequency. Next, 2 μL of sample was again gradually pipetted onto the chip, and this process was repeated until a total volume of 12 μL was applied (total run time of approximately 2 min). Resulting MS data was compiled into a single data file. The SAWN chip was cleaned with EtOH before applying new samples in order to prevent carryover and contamination. Representative blanks were run for testing purposes.

### MS Analysis

All SAWN experiments were performed on a TripleTOF 5600+ mass spectrometer (AB Sciex; Concord, ON, Canada) using a SAWN device and piezoelectric chip manufactured by Deurion (Seattle, WA, USA). Analyses were carried out at an interface heater temperature of 150 °C. Inlet and outlet gas pressures were both set to 0 psi, while the curtain gas pressure (nitrogen) was set to the system minimum (10 psi). Mass spectra (*m/z* 40–500) were acquired using multichannel acquisition (MCA) for 120 s, with an accumulation time of 3 s for a full spectrum at a resolution of just under 35,000 (at *m/z* 956). All samples were measured in negative ionization mode, with the exception of TATP and HMTD, which required positive ionization mode. The ISVF (ion spray voltage floating) was set to + 2200 V in positive mode and − 2200 V in negative mode. Data processing was done using PeakView 2.0 computer software (Sciex).

## Results and Discussion

This work reports for the first time the SAWN-MS analysis of organic explosives and the detection of nine frequently encountered energetic compounds in real forensic casework samples using this novel ambient ionization method. All identified peaks in the SAWN-MS spectra of stock solutions (1 μg/mL for nitroexplosives and 30 μg/mL for peroxides), their corresponding intensities, and suggested molecular structures are summarized in Table 1. In the following paragraphs, the SAWN ionization characteristics for the various explosive classes are discussed in more detail. The organic peroxide explosives were ionized and analyzed in positive mode and all the nitro-based explosives in negative mode.

**Table 1:** Suggested ions and their corresponding intensities in the SAWN-MS spectra of nine organic explosives

Explosive class	Explosive	Molecular weight (g/mol)	Observed ions *m/z* (intensity %)	Suggested ion
Peroxide-based (30 μg/mL)	TATP C_9_H_18_O_6_	222.24	149.0821 (13)* 166.1084 (25)* 240.1463 (17)* 247.0813 (32)* 261.1375 (8)* 279.1078 (55)*	[DADP + H]^+^ [DADP + NH_4_]^+^ [M + NH_4_]^+^ [C_8_H_16_O_7_ + Na]^+^ [M + K]^+^ [C_9_H_20_O_8_ + Na]^+^
	HMTD C_6_H_12_N_2_O_6_	208.07	145.0615 (18)* 179.0652 (10)* 209.0779 (7)* 229.0446 (63)* 245.0194 (22)*	[C_5_H_9_N_2_O_3_]^+^ [M + H-CH_2_O]^+^ [M + H]^+^ [M-2H + Na]^+^ [M-2H + K]^+^
Nitrate-esters (1 μg/mL)	PETN C_5_H_8_N_4_O_12_	316.14	315.0007 (3)* 350.9779 (18)* 352.9750 (5)* 377.9975 (57)* 428.9951 (100)*	[M-H]^−^ [M + ^35^Cl]^−^ [M + ^37^Cl]^−^ [M + NO_3_]^−^ [M + TFA–H]^−^
	ETN C_4_H_6_N_4_O_12_	302.11	318.9975 (3) 336.9656 (19) 338.9611 (8) 363.9852 (100) 369.9969 (63)	[M-NO_2_ + HNO_3_]^−^ [M + ^35^Cl]^−^ [M + ^37^Cl]^−^ [M + NO_3_]^−^ [ETriN+TFA-H]^−^
	NG C_3_H_5_N_3_O_9_	227.09	261.9833 (13) 263.9799 (5) 289.0033 (46) 302.0241 (11) 340.0022 (100)	[M + ^35^Cl]^−^ [M + ^37^Cl]^−^ [M + NO_3_]^−^ [M + CH_2_ONO_2_–H]^−^ [M + TFA–H]^−^
Nitroaromatics (1 μg/mL)	TNT C_7_H_5_N_3_O_6_	227.13	226.0064 (100) 227.0099 (8)	[TNT–H]^−^ [TNT]^−^
	Tetryl C_7_H_5_N_5_O_8_	287.15	227.9862 (24) 241.0171 (9) 318.0295 (13) 321.9797 (8) 348.9998 (65) 350.0017 (6) 399.9978 (100)	[N-methylpicramide-CH_2_]^−^ [N-methylpicramide-H]^=^ [M + NO]^−^ [M + ^35^Cl]^−^ [M + NO_3_]^−^ [M + NO_2_ + OH]^−^ [M + TFA–H]^−^
Nitroamines (1 μg/mL)	RDX C_3_H_6_N_6_O_6_	222.12	256.9988 (10) 258.9965 (4) 284.0180 (11) 335.0160 (100)	[M + ^35^Cl]^−^ [M + ^37^Cl]^−^ [M + NO_3_]^−^ [M + TFA–H]^−^
	HMX C_4_H_8_N_8_O_8_	296.16	331.0277 (100) 333.0250 (34) 341.0569 (16) 358.0473 (52) 359.0486 (4) 385.0841 (5) 409.0462 (90)	[M + ^35^Cl]^−^ [M + ^37^Cl]^−^ [M + NO_2_-H]^−^ [M + NO_3_]^−^ [M + NO_2_ + OH]^−^ [M + 3NO]^−^ [M + TFA–H]^−^

*Results that were achieved with the use of an additive (see “Results and Discussion” section for details)

### SAWN-MS Analysis of Organic Peroxide Explosives

The organic peroxides TATP and HMTD are of high priority due to their frequent use by terrorists and criminals. These compounds can be synthesized in a relatively simple manner but also easily detonate and are friction sensitive. Over the past few decades, peroxides were involved in several deadly explosives attacks, including more recently the Paris (2015) and Brussels attacks (2016), and the Manchester Arena bombing of 2017. Unfortunately, these compounds are also difficult to detect and analyze with MS due to lack of nitrate groups and the excessive fragmentation that can occur during ionization. Despite these difficulties, ESI-MS analysis of TATP has been reported using ammonium acetate as an additive, which enhances sensitivity of the method through the formation of ammonium adducts [55]. Additives can also provide a more robust and reproducible ion adduct formation by reducing the effect of instrument contaminations on analyte ionization [56].

The additives explored for the SAWN-MS analysis of the peroxide-based explosives were ammonium acetate (5, 10, 20, 50 mM), ammonium hydroxide (7, 15, 30%), and formic acid (0.1, 0.5%). Of these three species, ammonium hydroxide yielded the highest molecular ion intensities for the analysis of TATP. This prompted further investigation into the analysis of different concentrations of ammonium hydroxide in water in combination with the organic solvents IPA, MeOH, and ACN. Optimal results for the SAWN-MS analysis of TATP were obtained with a mixture of 85 *v*% IPA and 15 *v*% of an aqueous ammonia solution (32 m%). This conclusion was based on the inspection of the mass spectra and the monitoring of two peaks in particular; *m/z* 240 and *m/z* 223 corresponding to [M + NH_4_]^+^ and [M + H]^+^, respectively.

**Figure 2:**
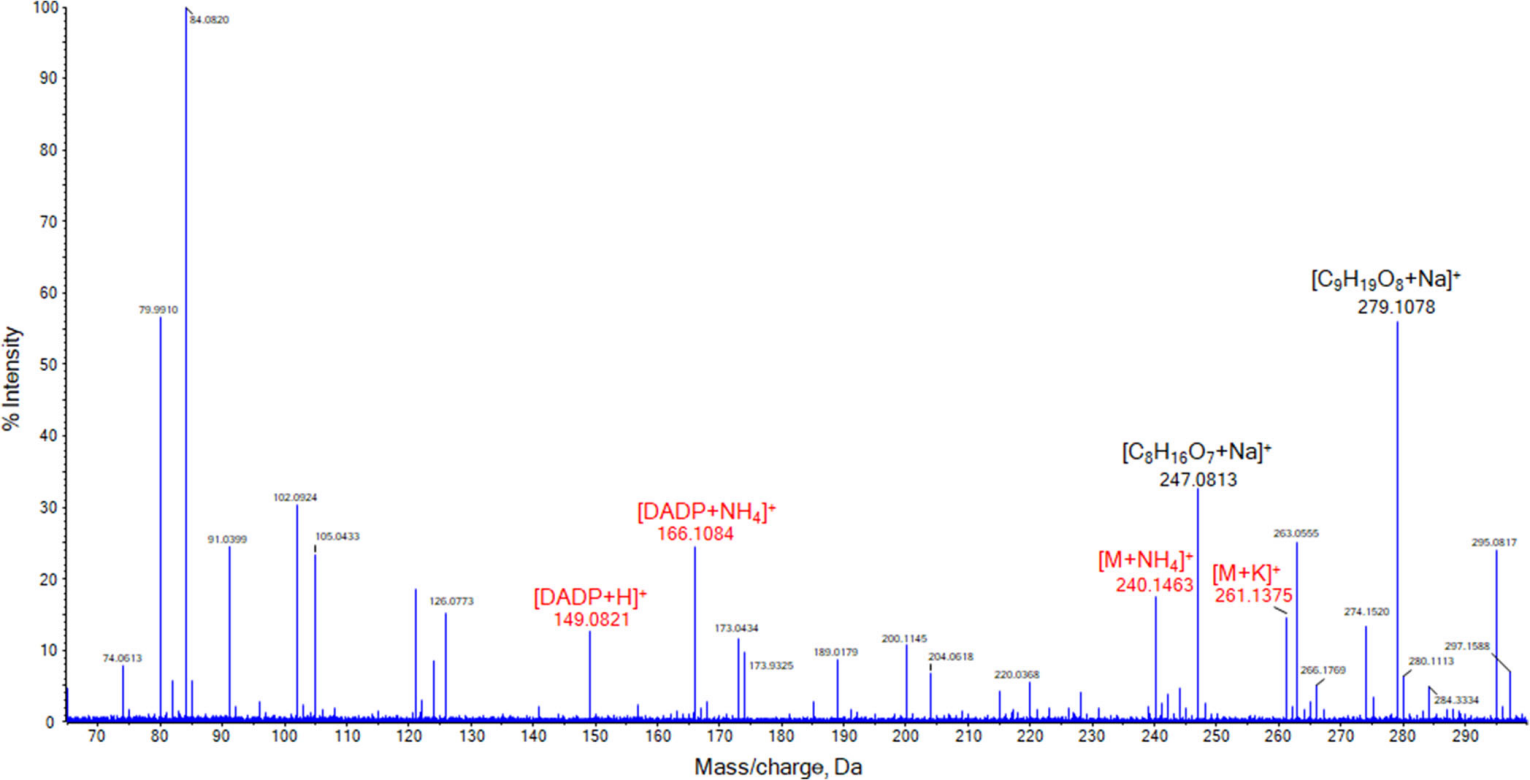
SAWN-MS spectrum of 30 μg/mL TATP in IPA/H_2_O (85:15 *v*/*v*%) with 15 *v*% NH_4_OH in aqueous phase, analyzed in positive ionization mode. Peaks labeled in black were identified as characteristic fragments, while those in red were identified as adducts of TATP and DADP, the analyte ion and degradation product, respectively

The SAWN-MS spectrum obtained for a 30 μg/mL TATP standard as depicted in Figure 2 shows extensive fragmentation and complex ion chemistry. This negatively affects sensitivity and hampers straightforward chemical identification. Identification of TATP was therefore based on adduct ions that directly relate to the analyte. More abundant ions are observed at *m/z* 247.0813 (attributed to [C_8_H_16_O_7_ + Na]^+^) and *m/z* 279.1078 (corresponding to [C_9_H_19_O_8_ + Na]^+^), both TATP-related ions that have been previously reported for NaCl doped ESI-MS and DESI-MS analysis [55, 57]. In addition, the signal at *m/z* 261.1375 ([M + K]^+^) is consistent with the MS analysis of TATP in the presence of potassium [19]. There are two peaks present at *m/z* 149.0821 and *m/z* 166.1084, which can be attributed to, respectively, the protonated ion and ammonium adduct of diacetone diperoxide (DADP), a degradation product of TATP. With direct MS analysis without chromatography, it is difficult to establish whether DADP is already present in the standard, is formed in the sample solution or is created during the nebulization and ionization processes. Nonetheless, the presence of DADP ions can also be considered as a strong indicator for the presence of TATP. Different concentrations of TATP were examined in order to qualitatively establish the limit of detection (LOD). At concentrations below 15 μg/mL, signal intensities of the analyte ions were generally too low to be distinguished from the noise level (the SAWN-MS spectrum of TATP at 15 μg/mL is depicted in Figure S1 of the Supplemental Information). Hence, this concentration was set as the detection limit as indicated in Table 2 which provides an overview of the LOD values for all explosive compounds studied.

**Table 2:** Experimental limits of detection (LODs) for all organic explosives investigated in this work (Reported LOD values are estimates based on a dilution series and correspond to the lowest concentration for which the main adduct ions for the explosive compound could still be clearly discerned from the background noise)

Explosive class	Explosive standard	Experimental LOD (ng/mL)
Nitrate esters	ETN	1
	PETN	10
	NG	10
Nitroamines	HMX	1
	RDX	1
Nitroaromatics	TNT	10
	Tetryl	10
Peroxide-based	HMTD	30,000
	TATP	15,000

**Figure 3:**
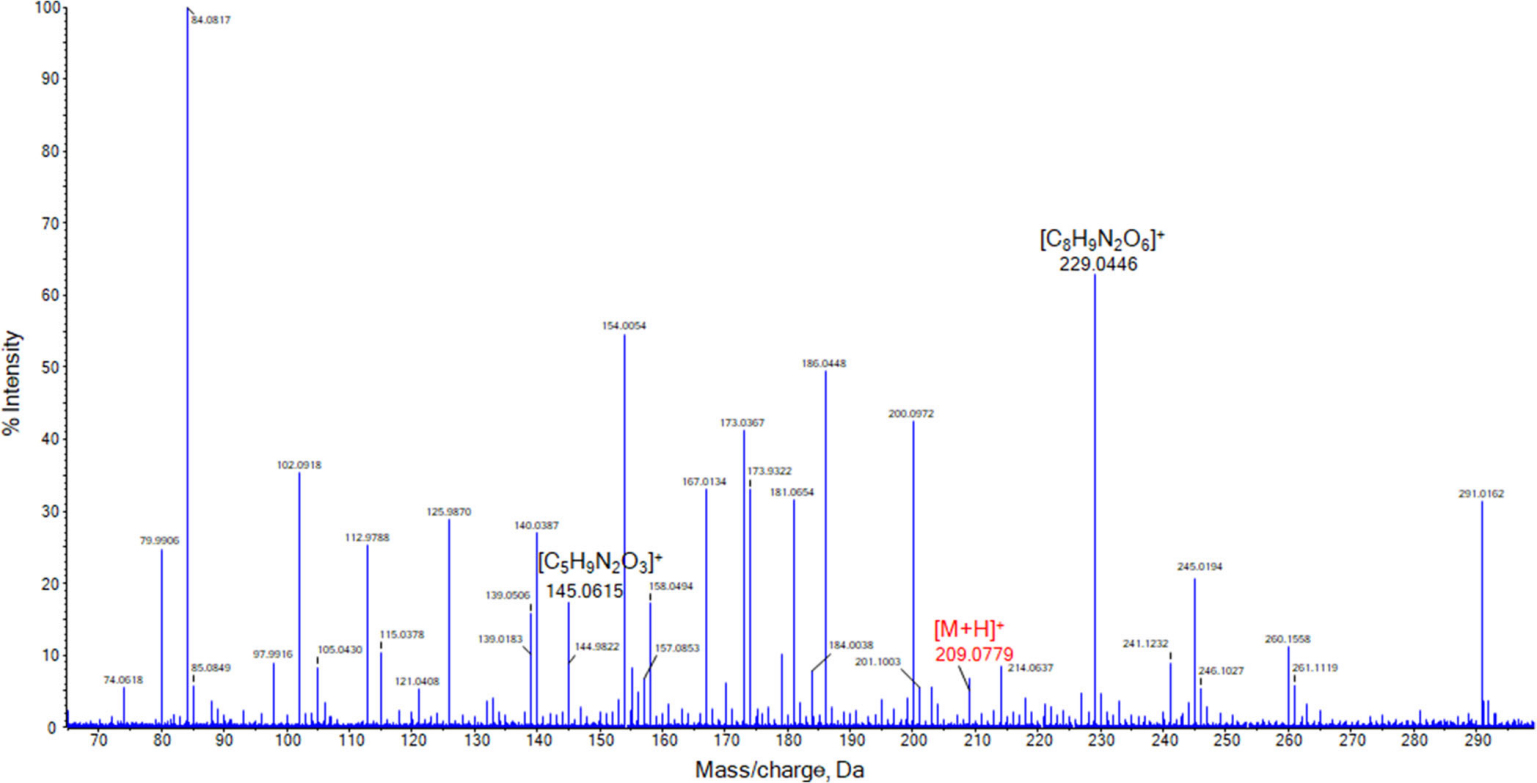
SAWN-MS spectrum of 30 μg/mL HMTD in IPA/H_2_O (85:15 *v*/*v*) with 0.1 wt% formic acid in aqueous phase, analyzed in positive ionization mode. Peaks labeled in black were identified as characteristic fragments of the analyte, while those in red were identified as adducts of HMTD

The investigation into optimum solvent conditions for the analysis of HMTD focused on the use of formic acid (0.1%, 0.5%) versus ammonium hydroxide, since formic acid has been shown to enhance the detection of this peroxide explosive [58]. HMTD peaks targeted for chemical identification were *m/z* 209 ([M + H]^+^) and *m/z* 179 ([M + H-CH_2_O]^+^). IPA with the addition of 15 *v*% of an aqueous solution of 0.1 wt% of formic acid was found to be the optimal solvent mixture. Despite the limited sensitivity observed, all ions that have been previously reported with ESI and DART [15, 59, 60] are also seen in the SAWN MS spectrum depicted in Figure 3. This spectrum was recorded for a 30 μg/mL solution of HMTD, and this concentration also corresponded to the detection limit as at lower levels, the key adduct ions could not be consistently differentiated from the background noise. The molecular ion of HMTD (*m/z* 209.0779) was observed with an intensity of only 7% relative to the base peak at *m/z* 84.0817. The peak at *m/z* 145.0615 represents the loss of hydrogen peroxide from this [C_5_H_11_N_2_O_5_]^+^ ion, as reported elsewhere [58, 60]. At *m/z* 229.0446 and *m/z* 245.0194, the loss of two hydrogen atoms from [M + Na]^+^ and [M + K]^+^, respectively, are seen with similar intensities as observed with laser electrospray [61].

Detection limits of 15 ppm and 30 ppm for TATP and HMTD, respectively, are clearly insufficient for the analysis of post-explosion samples were residual concentrations of explosives can be in the low ppb range. So currently, the presented SAWN-MS method can only be used for the analysis of intact peroxide explosive material. More tailor-made chemistry will be required to be able to confidently analyze post-explosion swabs for the presence of peroxide-based explosives with SAWN-MS in positive mode.

### SAWN-MS Analysis of Nitrate Ester Explosives

The nitrate esters are among the most frequently encountered explosives in forensic casework in the Netherlands [62]. They are part of military and civil grade explosives but are also found in IEDs (improvised explosive devices) and are used to commit crimes such as ATM raids often causing significant damage to the infrastructure. Three nitrate ester explosives were investigated in this study; PETN, ETN, and NG. MS methods commonly employed for the analysis of these explosives are based on APCI (atmospheric pressure chemical ionization) and ESI in negative mode with the addition of a chlorinated species to promote the formation of negatively charged adducts [56, 63, 64]. For this reason, different concentrations of chloroform were investigated in this study as a potential additive to enhance signal intensities of the analyte. IPA, MeOH, and ACN were used in various compositions in order to determine the ideal solvent mixture for analysis.

**Figure 4:**
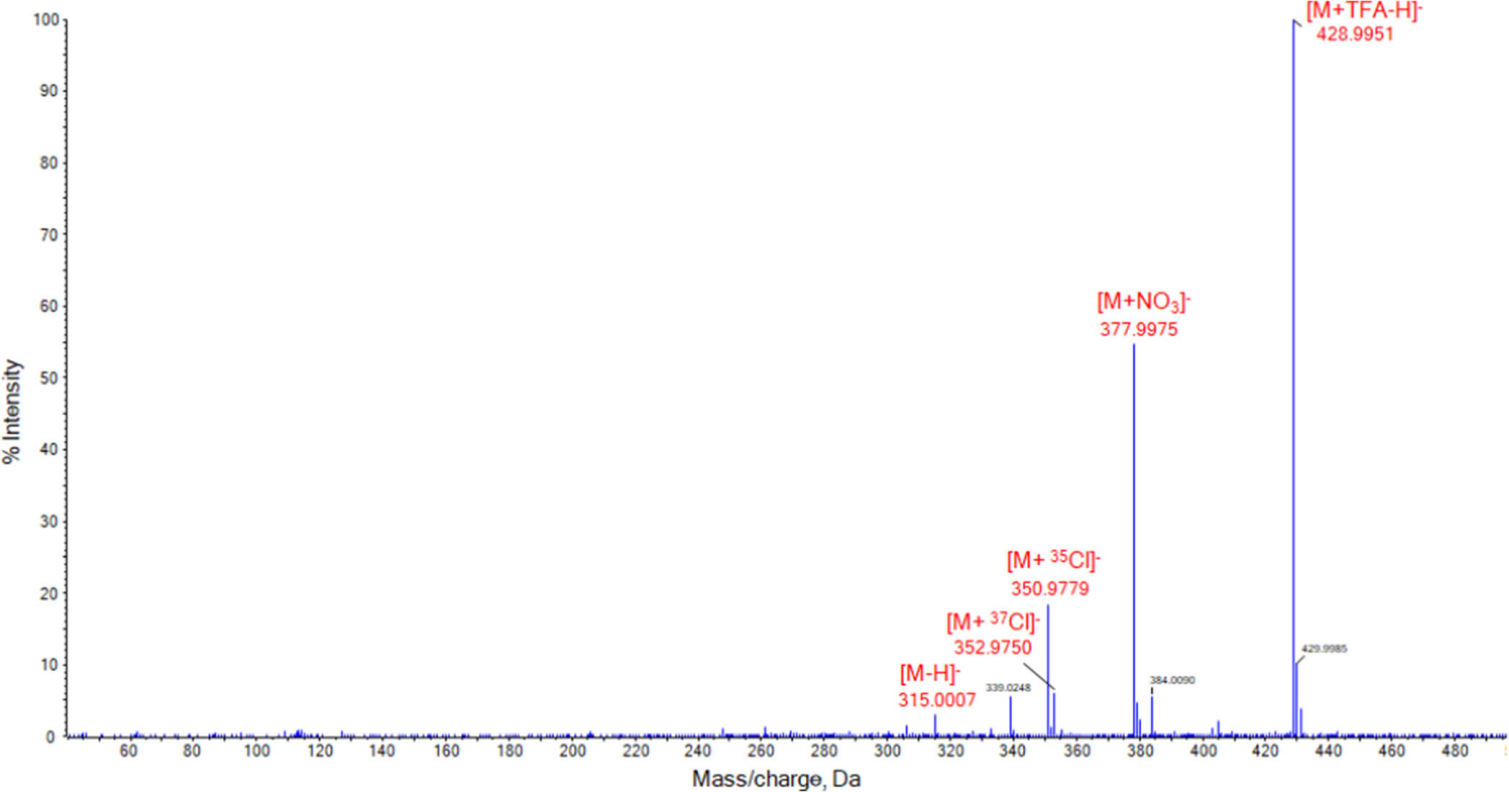
SAWN-MS spectrum of 1 μg/mL PETN diluted in MeOH/H_2_O (70:30 *v*/*v*%) + 0.1 *v*% CHCl3, analyzed in negative ionization mode. Peaks labeled in red were identified as adducts of PETN

Unlike the peroxide-based explosives, MeOH proved to be the better solvent choice, offering higher signal intensities and reduced baseline noise. For PETN, the optimum solvent mixture was found to be MeOH/H_2_O (70:30 *v*/*v*%). In terms of chloroform addition, ion intensities decreased with increasing chloroform concentration, and hence, all samples were prepared using 0.1 *v*% CHCl_3_. The peaks monitored for solvent optimization were *m/z* 378, *m/z* 351, and *m/z* 315, corresponding to [M + NO_3_]^−^, [M + ^35^Cl]^−^, and [M–H]^−^, respectively. The spectrum obtained from the analysis of 1 μg/mL PETN is depicted in Figure 4. Surprisingly, the most abundant (base) peak was determined to be a TFA adduct ([M + TFA–H]^−^). TFA was not added to the samples as an adduct agent but the source probably originates from the MS instrument as this compound is used extensively in mobile phases for bio-LC-MS analysis on this setup. In this study, TFA adduct ions were observed for all nitroexplosives with the exception of TNT. Hence, this adduct was also considered as a characteristic ion for the identification of nitroexplosives including PETN. The nitrate adduct, generated through ionization in air (the explosive compound provides its own source of nitrate), is observed at *m/z* 377.9975, as previously reported using low temperature plasma desorption [65]. At *m/z* 350.9779 and *m/z* 352.9750, the chlorinated ions were detected, albeit at lower relative intensities than reported using LC-MS and APCI [56, 63, 66]. Finally, the deprotonated molecular ion [M–H]^−^ is seen at *m/z* 315.007 in line with reported ESI-MS results [63].

Dilutions of PETN were made in order to estimate the LOD of this explosive. The ions identified at 1 μg/mL are all present and clearly distinguishable from the noise level down to a concentration of approximately 10 ng/mL (see Table 2). At this concentration, the TFA adduct remains the most abundant ion, while the nitrate and chloride ion adducts all exhibit a decrease in intensity, as depicted in Figure S2 (Supplemental Information).

Unlike PETN, pure MeOH was found to offer the best sensitivity for ETN. No additive was needed for the formation of analyte ions (including chloride adducts) with intensities similar to those documented using APCI [67]. The SAWN-MS spectrum depicted in Figure S3 of the Supplemental Information shows the two Cl adduct isotopes at *m/z* 336.9565 and *m/z* 338.9611. Consistent with results obtained using DART, the most intense signal corresponds to [M + NO_3_]^−^, while [M–NO_2_ + HNO_3_]^−^ is present at *m/z* 318.9975 [68]. Just as with PETN, an adduct is formed with TFA. However, in this case, the adduct is predominantly formed with erythritol trinitrate (ETriN), a degradation product of ETN that could be formed during the ionization process. The sensitivity for the detection of ETN proved to be better than that of PETN, with an observed LOD of approximately 1 ng/mL. The chloride adducts at this concentration are just above noise level, while the intensity of [M + NO_3_]^−^ drops to approximately 40%. Interestingly, at this trace level, the TFA adduct with ETN now yields the most abundant signal in the spectrum (see Figure S4 of the Supplemental Information).

NG in MeOH (1 μg/mL) was analyzed by SAWN-MS, the resulting spectrum is displayed in Figure S5 of the Supplemental Information. Chloroform was required to obtain the [M + Cl]^−^ ion at *m/z* 261.9833. The nitrate adduct (*m/z* 289.0033) reaches an intensity of 46%, although with ESI and APCI, higher intensities have been reported for this ion [56, 66]. In line with PETN, the most intense peak was attributed to [M + TFA–H]^−^. Additionally, the [M + CH_2_ONO_2_–H]^−^ ion is seen at *m/z* 302.0241, consistent with results published by Xu et al. [69]. Like PETN, a LOD was estimated of 10 ng/mL. The nitrate and TFA adducts are observed as the most intense signals at this concentration (Figure S6 of the Supplemental Information).

### SAWN-MS Analysis of Nitroamine Explosives

**Figure 5:**
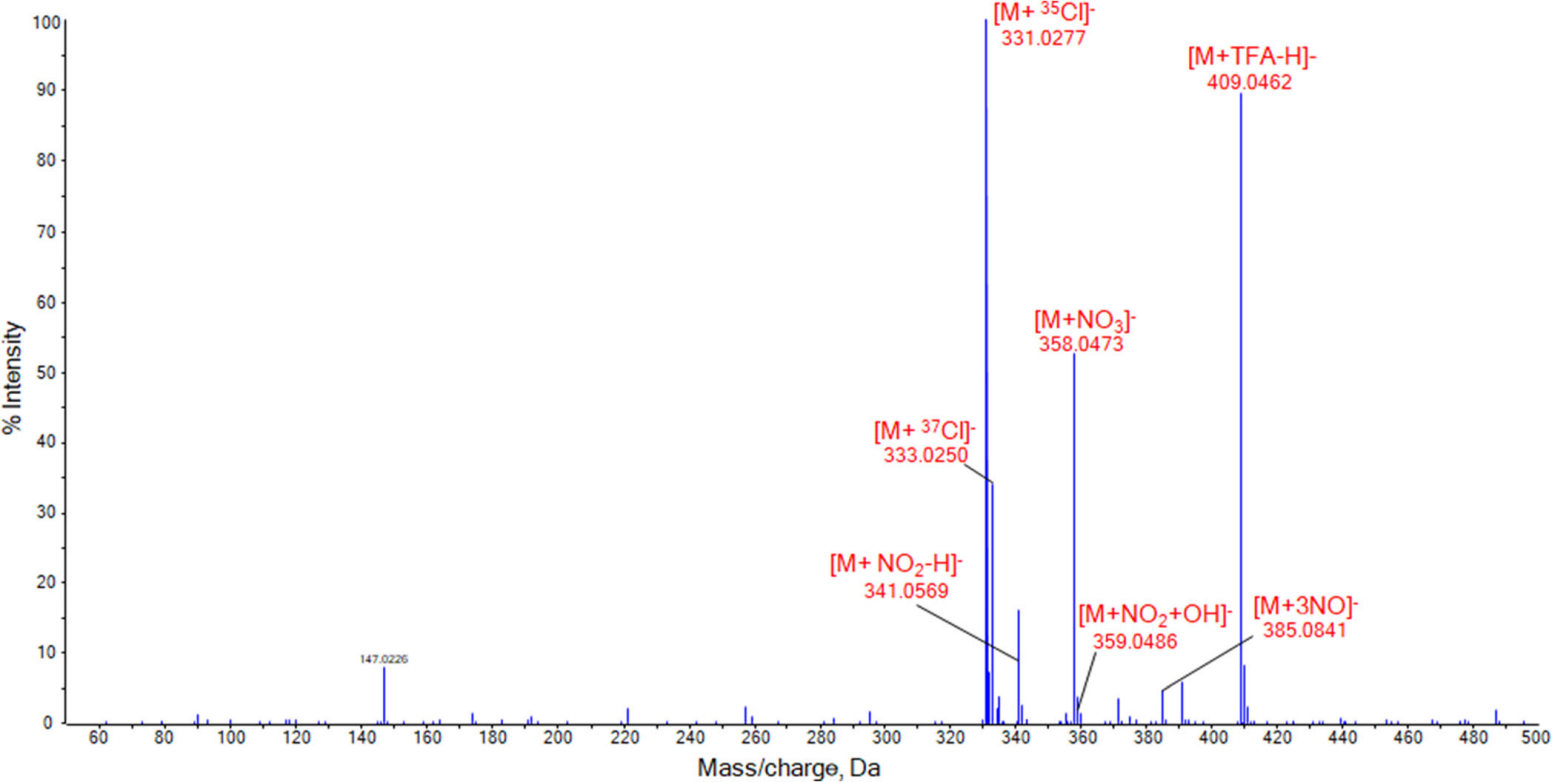
SAWN-MS spectrum of 1 μg/mL HMX in MeOH, analyzed in negative ionization mode. Peaks labeled in red were identified as adducts of RDX

The analysis of nitroamines has been a topic of great interest in forensics due to the military and civilian applications of these high explosives [70]. RDX and HMX are often used in the preparation of “plastic” explosives and mixtures. The SAWN-MS analysis of RDX and HMX proved to be rather straight forward. Excellent sensitivity and spectral consistency were obtained using pure MeOH as the solvent. At a concentration of 1 μg/mL all peaks in the resulting spectrum of RDX could be attributed to the analyte as illustrated in Figure S7 (Supplemental Information). The most abundant signal (*m/z* 335.0160) corresponds to an adduct formed with TFA. The [M + ^35^Cl]^−^ ion is observed at *m/z* 256.9988 at an intensity of 10% and the [M + NO_3_]^−^ ion at *m/z* 284.0180. These results are consistent with findings reported in literature [63]. Most peaks in the SAWN-MS spectrum of HMX could be directly assigned to the compound of interest as shown in Figure 5. The ions at *m/z* 331.0277 and *m/z* 333.0250 represent [M + ^35^Cl]^−^ and [M + ^37^Cl]^−^, respectively, and are in agreement with results reported for LC-ESI-MS [71]. TFA present in the system again forms an adduct with the analyte, yielding a signal at *m/z* 409.0462 at a relatively high intensity. The nitrate adduct at *m/z* 358.0473 is observed at an intensity of 52%. Other characteristic peaks for this explosive are observed at *m/z* 385.0841 ([M + 3NO]^−^), *m/z* 359.0486 ([M + NO_2_ + OH]^−^), and *m/z* 341.0569 ([M + NO_2_–H]^−^) in line with published findings using ESI [72]. The two nitroamine standards were diluted in MeOH in order to investigate the sensitivity of the SAWN-MS analysis. The nitrate, chloride, and TFA adduct of RDX and HMX could all be identified at concentrations as low as 1 ng/mL as shown in Figures S8 and S9 of the Supplemental Information, respectively.

### SAWN-MS Analysis of Nitroaromatic Explosives

**Figure 6:**
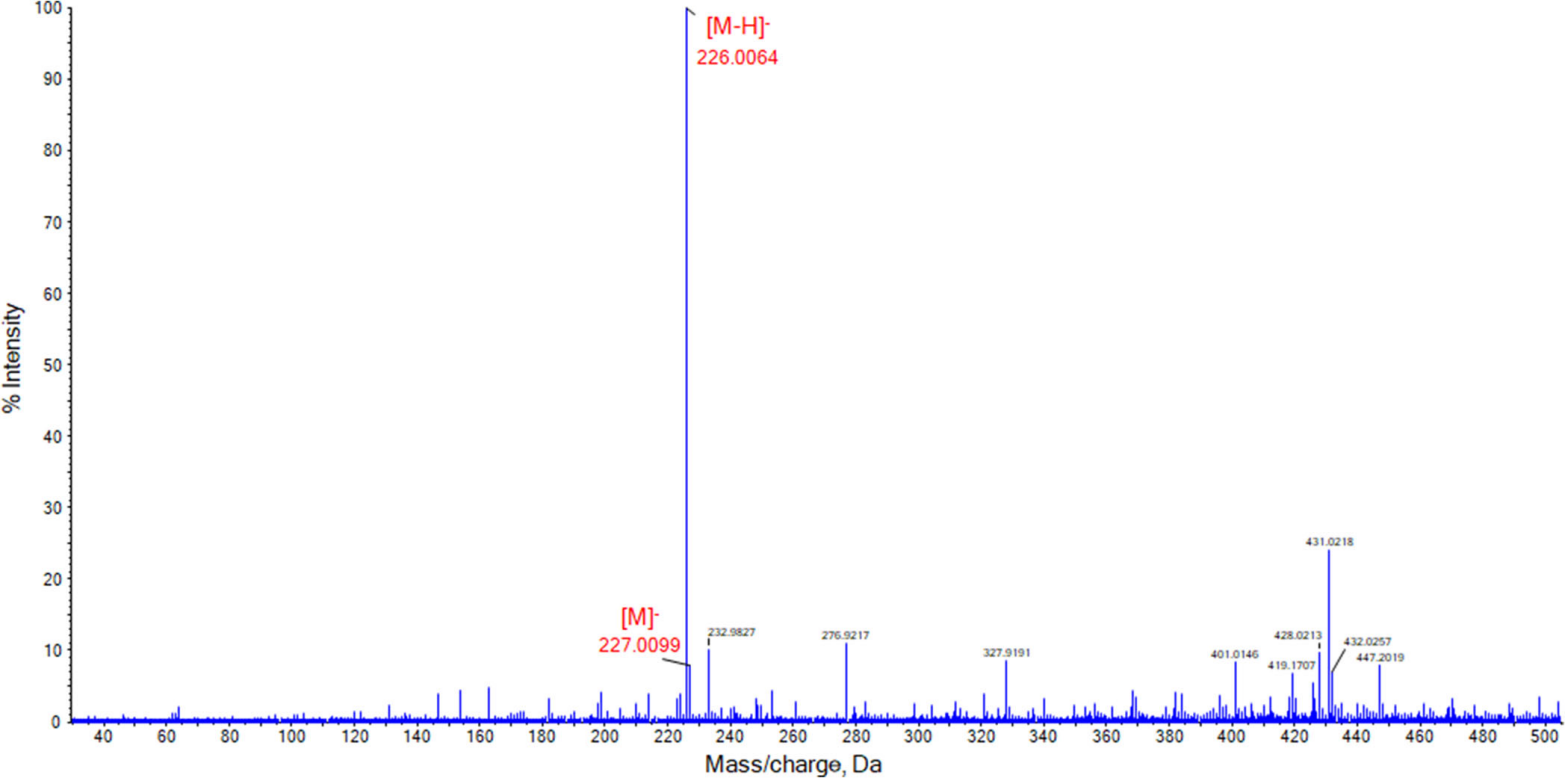
SAWN-MS spectrum of a 1 μg/mL TNT in MeOH, analyzed in negative ionization mode. Peaks labeled in red were identified as TNT-related ions

Nitroaromatic explosives have been used for military and civil purposes for decades. TNT is known as a high explosive with excellent stability and safety features [70]. Nitroaromatics are typically encountered in forensic case work when crimes are committed with illegally obtained military explosives like hand grenades or rocket-propelled grenades (RPGs). This study includes the analysis of two nitroaromatic compounds: TNT and tetryl. The SAWN-MS spectrum of a 1 ppm (1 μg/mL) solution of TNT in MeOH is depicted in Figure 6. The intensities of both identified ions are consistent with results obtained using LC-APCI-MS [69]. The [M–H]^−^ ion is detected at *m/z* 226.0064, while [M]^−^ is observed at *m/z* 227.0099. The highest intensity (100%) for the molecular ion is in line with reported findings for other ambient ionization techniques such as APCI, ESI, and APPI [63, 65, 72–75]. Initial testing determined that MeOH/H_2_O (70:30 *v*/*v*%) was required for successful ionization of tetryl, and all subsequent samples were therefore prepared in this solvent mixture.

Figure S10 (Supplemental Information) shows the 1 ppm SAWN-MS spectrum of tetryl. The nitrate adduct [M + NO_3_]^−^, present at *m/z* 348.9998, has not been reported before in ESI and LC-APCI studies [69, 76, 77]. In addition, the [M + Cl]^−^ ion (*m/z* 321.9797) was successfully identified, albeit at a low relative intensity (7%). The peaks at *m/z* 241.0171 and *m/z* 227.9862 represent adducts of N-methylpicramide, the hydrolysis product of tetryl. The [M + TFA–H]^−^ adduct represents the base peak at 100% intensity. Other identified analyte peaks include [M + NO]^−^ (*m/z* 318.0295) and [M + NO_2_ + OH]^−^ (*m/z* 350.0017). The LOD observed for both nitroaromatic species was estimated at 10 ng/mL. Trace level SAWN-MS spectra for Tetryl and TNT are shown in Figures S11 and S12 in the Supplemental Information, respectively.

**Figure 7:**
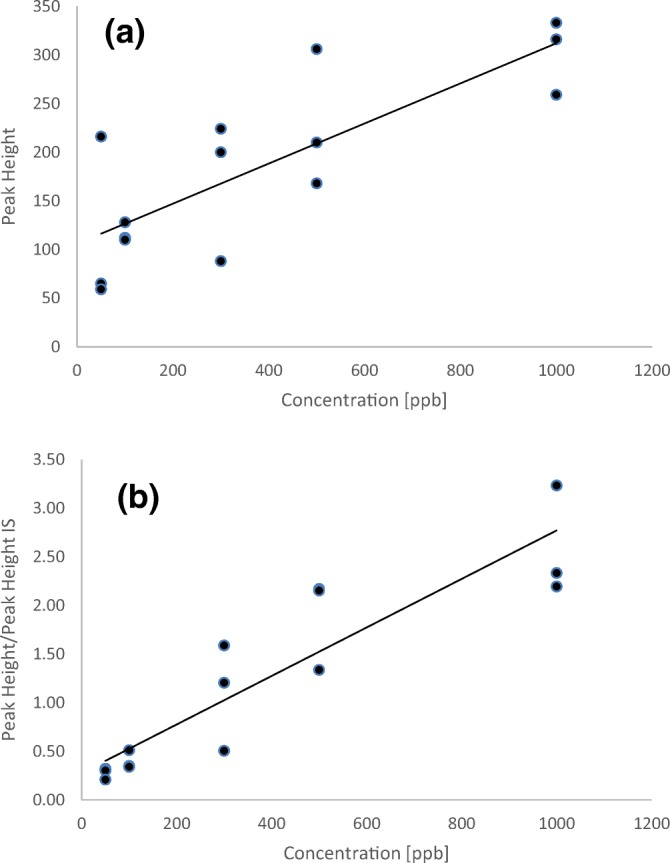
Calibration curves for TNT by plotting the peak height as such as function of concentration (**a**), and by plotting the peak height ratio versus benzoic acid as internal standard (**b**)

The detection limits reported in Table 2 through application of a dilution series are of an indicative nature. The more regular approach by constructing a calibration curve and establishing the LOD from the sensitivity (slope of the curve) and the baseline noise is not easily implemented with SAWN-MS as the amount of analyte introduced and ionized in the mass spectrometer is uncontrolled and highly variable. This could be further improved through precise microvolume dosing but controlling the nebulization process will remain challenging. Although quantitative analysis is usually less important in forensic explosive analysis, the use of an internal standard (IS) could provide options similar to drug analysis with PS-MS [78]. This was demonstrated for TNT by using benzoic acid as an internal standard. The ideal IS typically is a deuterated form of the analyte of interest but such standards are not commercially available for explosives. Therefore, benzoic acid was chosen as an internal standard as it has some structural resemblance to TNT and yields a straightforward SAWN-MS spectrum with an abundant [M–H]^−^ ion at *m/z* 121.034. From a stock solution of 1 μg/mL in MeOH, dilutions of 500, 300, 100, and 50 ng/mL of TNT were prepared containing 1 μg/mL benzoic acid. Triplicate measurements were taken at each concentration, and the calibration curves shown in Figure 7 were constructed from the peak height for [TNT–H]^−^ and the peak height ratio versus benzoate. Some improvement can be seen when applying peak height ratios indicating that the IS can account for some of the variation in the SAWN-MS analysis. However, reproducibility of the measurements remains an issue and therefore affects sensitivity. A significant run-to-run variation in the LOD can be expected in the current SAWN setup and more work must be done in order to better control the nebulization and ionization.

### Analysis of Explosive Mixtures with SAWN-MS

Although the results described above are promising, it should be noted that any practical technique for the detection and identification of energetic materials must be able to analyze a wide range of explosives in a single method. Having multiple methods is suboptimal even if the instruments are portable and rapid. Furthermore, explosive charges can consist of mixtures of compounds, both energetic materials and additives. Well-known explosive mixtures include Semtex (RDX + PETN), Composition B (RDX + TNT), Octol (HMX + TNT), Pentolite (PETN + TNT), and C-4 (RDX). Some of these compounds are composed of only the explosives themselves, while others incorporate other materials such as organic fuels, taggants, plasticizers, binders, and oils in order to improve the traceability, performance, and physical features of the product.

**Figure 8:**
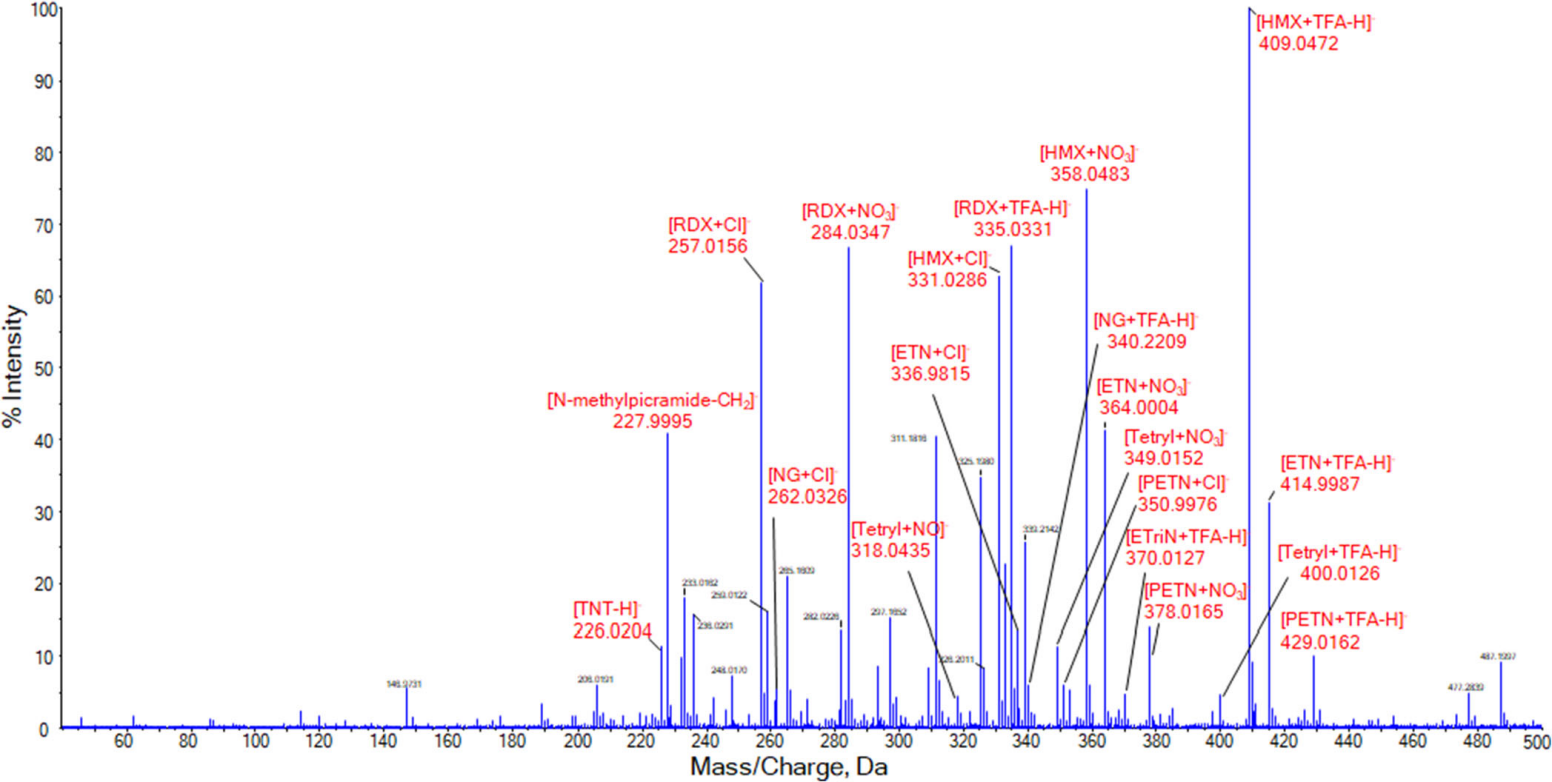
SAWN-MS spectrum of a mixture of PETN, ETN, NG, RDX, HMX, TNT, and tetryl, analyzed in negative ionization mode. Concentration of 1 μg/mL in MeOH + 0.1 *v*% CHCl_3_ for each compound. Peaks labeled in red were identified as analyte related (adduct) ions

To investigate whether SAWN-MS is able to identify multiple explosives in a single analysis, a mixture of every nitroexplosive was prepared. The final standard mixture consisted of PETN, ETN, NG, RDX, HMX, TNT, and tetryl, each at a concentration of 1 μg/mL in MeOH with 0.1 *v*% CHCl_3_ added. Figure 8 shows the corresponding SAWN-MS spectrum. The nitroamines (RDX and HMX) were both easily identified, as they represent the most abundant peaks in the spectrum. At 100% intensity, [HMX + TFA–H]^−^ but also the nitrate and chloride adducts of this component are detected. The same adducts can be identified for RDX. The three nitrate esters (ETN, PETN, NG) could also be successfully discerned. The nitrate adduct of ETN is present at a fairly high intensity, while the [ETN + Cl]^−^ ion is detected at a relative intensity of 14%. In addition to the [ETriN + TFA–H]^−^ ion, this time also a significant signal for the TFA adduct of ETN is observed. With respect to PETN, all adducts that are seen in the individual spectrum of the standard are also identified in the mixture analysis. NG proved to be harder to identify, with associated ions having an intensity of about 6% of the base peak in the mass spectrum of the mixture. Nevertheless, the chloride adduct can be detected just as the [NG + TFA–H]^−^ ion thus enabling robust identification of this nitrate ester. With respect to the nitroaromatics, ions of TNT and tetryl are both present in the mass spectrum. [Tetryl + TFA–H]^−^ is observed at 5% intensity, while N-methylpicramide can also be distinguished just as the nitrate and nitric oxid adducts. Finally, TNT is identified at nominal mass 226.

Hence, it can be concluded that all nitroexplosive species investigated in this study could be identified when analyzed as a mixture in a single SAWN-MS run. It is important to note that despite the multiple adducts formed, no isobaric overlap occurs for these compounds. The mass resolution of the TOF-MS system used in this study is high enough to prevent any overlap or misidentification of the explosives considered. If a mass spectrometer with unit mass resolution would be used in combination with SAWN ionization, care would have to be taken to distinguish the following ions that are within 1 Da mass difference: 227 (TNT)–228 (tetryl), 318 (tetryl)–319 (ETN), and 340 (NG)–341 (HMX) (for adduct details, see Table 1).

Surprisingly, ions of PETN and tetryl could be detected despite being diluted in MeOH, rather than MeOH/H_2_O, albeit at reduced sensitivity. While applying the dilution series as for the regular standards, all explosive compounds could be identified at an individual concentration of 500 ng/mL. While completely satisfactory for the investigation of intact material, this multicompound sensitivity could be insufficient when analyzing post-explosion traces.

### Analysis of Forensic Pre- and Post-Explosive Case Work Samples

To determine the true potential of novel techniques, the performance when analyzing real forensic case samples needs to be assessed. To this end, anonymous extracts from case work, both intact material solutions and post-explosion samples, were provided by the Netherlands Forensic Institute (NFI). To prevent bias with the research team, it was decided that the forensic experts would only share the results of the validated in-house LC-HRMS method [64] (for which the outcomes are considered as the ground truth) after the SAWN-MS experiments and data analysis were completed. By comparing results with the findings of the NFI, the sensitivity and selectivity of the direct analysis with SAWN-MS could be investigated under realistic conditions.

**Figure 9:**
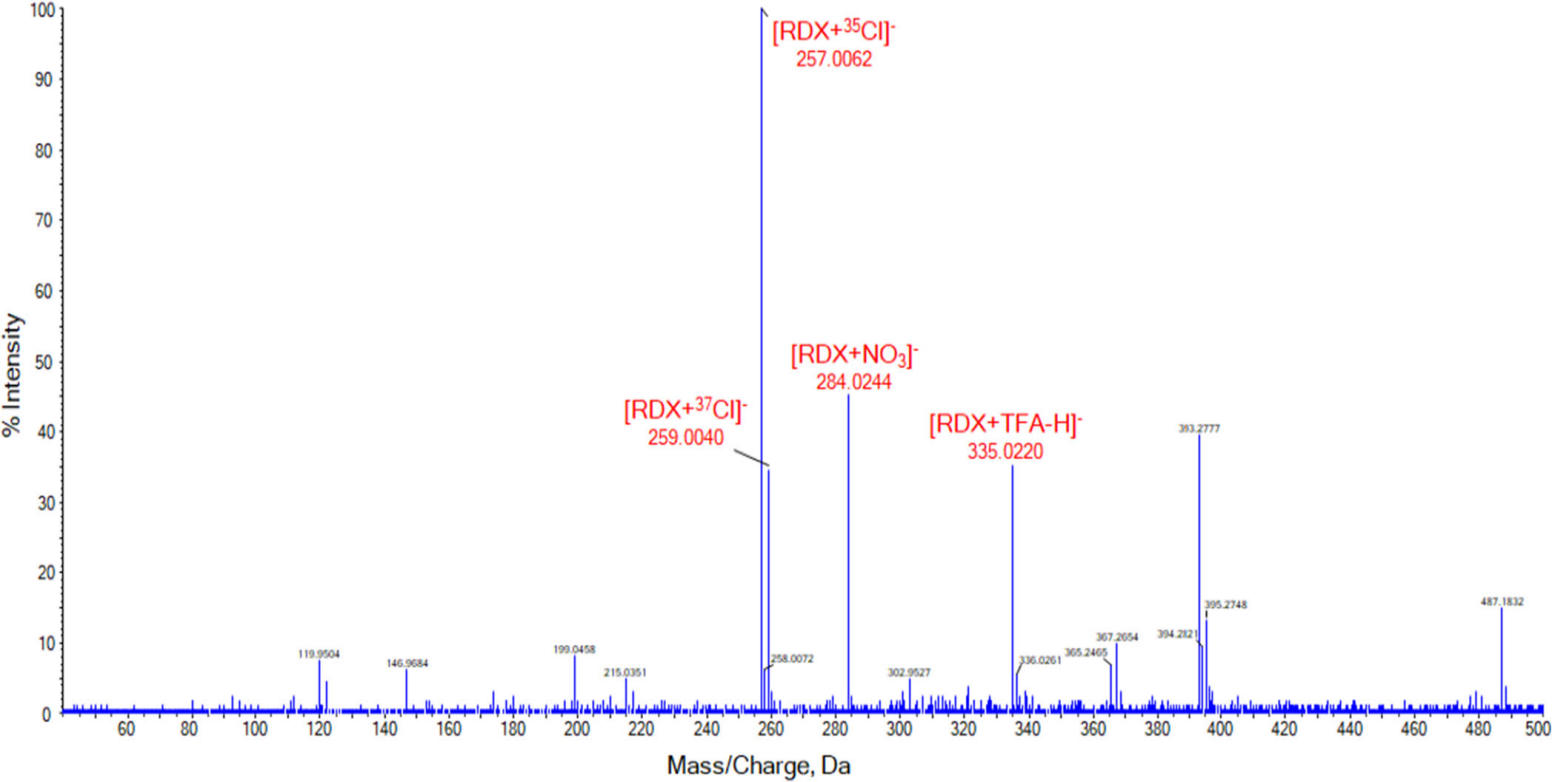
SAWN-MS analysis of forensic case extract 11 (post-explosion swab) showing the presence of RDX

In total, 14 case samples were supplied by the NFI, eight post-explosion/residue samples and six solutions of intact material. An overview of the case work samples, the SAWN-MS results and the LC-HRMS findings of the NFI is given in Table 3. Figure 9 illustrates the detection of RDX in a post-explosion forensic case extract with SAWN-MS. The remaining mass spectra for the positive samples are provided in the Supplementary Information (Figures S13–S20). With regard to the pre-explosion sample set, the correct explosive was identified for all six case samples, with high signal intensities for the same adduct ions as observed in the analysis of the respective standards. The spectra were collected after a 10-fold dilution of the case sample in MeOH.

**Table 3:** SAWN-MS analysis of case extracts provided by the NFI and comparison with LC-MS results using a validated and accreditedMethod at the forensic institute

Case sample no.	LC-MS results	Sample type	SAWN-MS results
1	~ 50 ng/μL PETN, ~ 200 ng/μL PETriN	Post-explosion swab	PETN
2	~ 1 ng/μL PETN, ~ 25 ng/μL PETriN	Post-explosion swab	No explosive detected (false negative)
3	~ 125 ng/μL TNT	Post-explosion swab	No explosive detected (false negative)
4	~ 100 ng/μL HMTD	Post-explosion swab	No explosive detected (false negative)
5	10 ng/μL TNT	Intact material	TNT
6	10 ng/μL RDX	Intact material	RDX
7	10 ng/μL PETN	Intact material	PETN
8	~ 30 ng/μL TNT	Post-explosion swab	TNT
9	~ 10 ng/μL PETN	Post-explosion swab	PETN
10	~ 3 ng/μL NG	Post-explosion swab	No explosive detected (false negative)
11	~ 3 ng/μL RDX	Post-explosion swab	RDX
12	100 ng/μL PETN	Intact material	PETN
13	100 ng/μL RDX	Intact material	RDX
14	100 ng/μL TNT	Intact material	TNT

Post-explosion case samples are collected after an explosion has occurred by swiping an area or object of interest with a MeOH wetted wipe and subsequent extraction of the residues from the wipe with additional MeOH. Such extracts are typically more challenging to analyze because the original explosive and associated degradation products are often present at trace (low ppb) level and because such extracts can contain matrix compounds that can negatively affect the analysis. Despite these challenges, four of the eight post explosion samples could be successfully analyzed using SAWN-MS yielding comparable results as obtained with LC-HRMS.

In total, ten of the fourteen case samples were correctly identified, with a 0% false positive rate. So, no SAWN-MS results showed the presence of an explosive that was not detected at the NFI. This indicates that contamination risks by using a single SAWN chip can be mitigated with a good cleaning protocol and blank runs in between sample runs. Regarding the solutions of intact explosives, this 0% false positive rate is combined with a 0% false negative rate, all samples were analyzed correctly on the basis of the LC-HRMS data. Unfortunately, for the post-explosion samples, a false negative rate of 50%, was observed. Four extracts yielding no result with SAWN-MS tested positive at the NFI for PETN, TNT, HMTD, and NG. For the four post-explosion extracts that were correctly analyzed with SAWN-MS, some samples required an addition of up to 2% CHCl_3_ in order to boost sensitivity. These results indicate that at this stage direct analysis with SAWN-MS is not sensitive and robust enough for post-explosion analysis. Given the relatively high concentrations reported for some of these extracts at the NFI, matrix induced ion suppression could also have negatively affected the sensitivity. Nevertheless, the analysis of intact energetic materials with SAWN-MS seems very promising, providing correct answers almost in real time.

## Conclusions and Future Work

The results presented herein illustrate a novel approach to the mass spectral analysis of organic explosives. SAWN-MS was investigated for the rapid and selective identification of nine organic explosives commonly encountered in forensic casework (TATP, HMTD, PETN, ETN, NG, RDX, HMX, TNT, and tetryl). Observed adduct ions with this relatively low-energy ambient ionization method were comparable to those reported using more widely implemented ambient ionization techniques, such as ESI and APCI. Not only is the proposed method easy to operate, but the cheap and simple setup allows for potential miniaturization into compact systems, ideal for coupling to portable mass spectrometers. In addition, analysis time is greatly reduced, minimal sample preparation is required, ionization and analysis take place in real-time and the ionization does not require external gas flows, heating or high voltages such as other ambient ionization techniques.

The sensitivity of explosives analysis with SAWN-MS was qualitatively explored in this work. Experimental limits of detection as low as 1 ng/mL were achieved for some of the nitroexplosives in negative mode, while the peroxide based explosives yielded much higher LODs in positive mode. However, the sensitivity of this system has yet to be fully investigated, understood, and optimized.

The use of benzoic acid as an internal standard was shown to significantly improve the calibration curve for the [M–H]^−^ ion of TNT, but reproducible analyte introduction and ionization remains challenging with SAWN. Although quantitative analysis is not a prerequisite in forensic explosives analysis, a better controlled nebulization and sample intake is still needed to ensure a reproducible LOD. This could possibly be achieved by accurate microdosing of liquids on the SAWN chip using microfluidic technology and by designing an appropriate enclosure. SAWN-MS was also suitable for analyzing mixtures of explosives as demonstrated with the identification of seven nitroexplosives of different classes in a single run. This feature is important for the use of SAWN-MS as a broad screening technique for organic explosives.

To test this new approach under realistic conditions, fourteen anonymized case work samples were analyzed. Very convincing results were obtained for intact material, the explosive compounds identified matched perfectly with the LC-HRMS results reported by the forensic laboratory. Although no false positive results were obtained, the limited sensitivity for some classes of explosive materials and the potential sensitivity loss due to matrix interference resulted in a 50% false negative rate for the post-explosion case extracts. Possibly, sensitivity could be increased further for the nitroexplosives by the addition of TFA to sample solutions as in the current work abundant signals were observed for TFA adduct ions due to TFA residues in the experimental setup.

Overall, SAWN-MS offers an attractive alternative to current methods used for the chemical identification of intact energetic materials but lacks robustness and sensitivity for some compounds of interest to be used for post-explosion investigations. The sensitivity of this technique could be better understood and potentially be further optimized to expand the application to large-scale on-scene post-explosion sample screening. This specifically applies to the analysis of peroxide explosives in positive mode for which currently detection limits in the ppm range are reported. In terms of selectivity and possibly sensitivity, the fragmentation of parent ions can be examined using tandem MS, which would allow for the identification of explosives in complex matrices. Such improvements would also provide opportunities for mass spectrometry as an alternative to ion mobility spectrometry for mass security screening in heavily trafficked areas (airports, arenas) to prevent terrorist attacks and other crimes involving explosives.

## Electronic Supplementary Material

ESM 1 (DOCX 548 kb)

## Abbreviations

ACN: Acetonitrile
APCI: Atmospheric pressure chemical ionization
CID: Collision-induced dissociation
DART: Direct analysis in real time
DESI: Desorption electrospray ionization
ETN: Erythritol tetranitrate
HMTD: Hexamethylene triperoxide diamine
HMX: 1,3,5,7-Tetranitro-1,3,5,7-tetrazocane
IED: Improvised explosive device
IMS: Ion mobility spectrometry
IDT: Interdigitated transducer
IPA: Isopropyl alcohol
MeOH: Methanol
MS: Mass spectrometry
NG: Nitroglycerin
PETN: Pentaerythritol tetranitrate
PS: Paper spray
RDX: Cyclotrimethylenetrinitramine
SAW(N): Surface acoustic wave (nebulization)
TATP: Triacetone triperoxide
TFA: Trifluoro acetic acid
Tetryl: Trinitrophenylmethylnitramine
TNT: 2,4,6-Trinitrotoluene
