# Supplementary material for: Utilizing Surface Acoustic Wave Nebulization (SAWN) for the Rapid and Sensitive Ambient Ionization Mass Spectrometric Analysis of Organic Explosives
Source: J Am Soc Mass Spectrom. 2019 Oct 28;30(12):2655–69. doi: 10.1007/s13361-019-02335-y (PMC6914713; doi:10.1007/s13361-019-02335-y)
Supplement: Supplementary file 1 — (DOCX 548 kb) [file 13361_2019_2335_MOESM1_ESM.docx]

**Utilizing surface acoustic wave nebulization (SAWN) for the rapid and sensitive ambient ionization mass spectrometric analysis of organic explosives**

Lauren Pintabona^1^, Alina Astefanei^1^, Garry L. Corthals^1 *^, Arian C. van Asten^1,2 *^

^1^ van ‘t Hoff Institute for Molecular Sciences, Faculty of Science, University of Amsterdam, PO Box 94157, 1090 GD Amsterdam, The Netherlands

^2^ CLHC, Amsterdam Center for Forensic Science and Medicine, University of Amsterdam, P.O. Box 94157, 1090 GD Amsterdam, The Netherlands

* corresponding authors

**Supplemental Information**


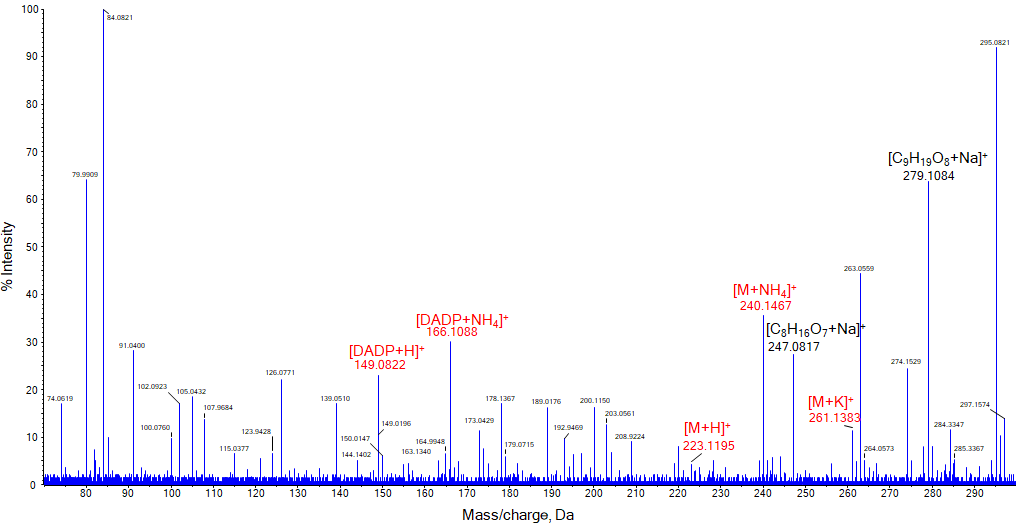


Figure S1 SAWN-MS spectrum of 15 µg/mL TATP in IPA/H_2_O (85:15 v/v%) with 15 v% NH_4_OH in aqueous phase, analyzed in positive ionization mode. Peaks labeled in black were identified as characteristic fragments, while those in red were identified as adducts of TATP and DADP, the analyte ion and degradation product, respectively.


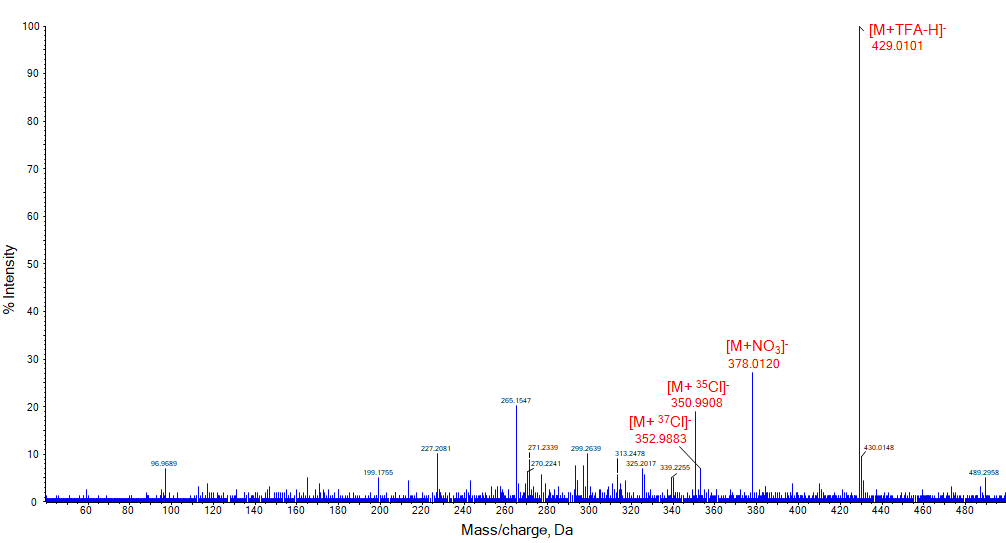


**Figure S2** SAWN-MS spectrum of 10 ng/mL PETN diluted in MeOH/H2O (70:30 v/v) + 0.1 v% CHCl3, analyzed in negative ionization mode. Peaks labeled in red were identified as adducts of PETN.


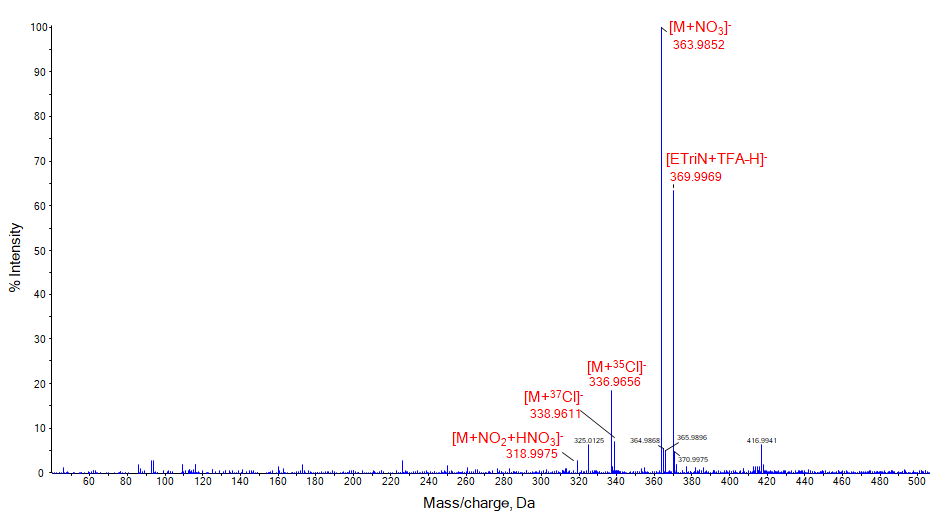


Figure S3 SAWN-MS spectrum of 1 μg/mL ETN in MeOH, analyzed in negative ionization mode. Peaks labeled in red were identified as adducts of ETN.


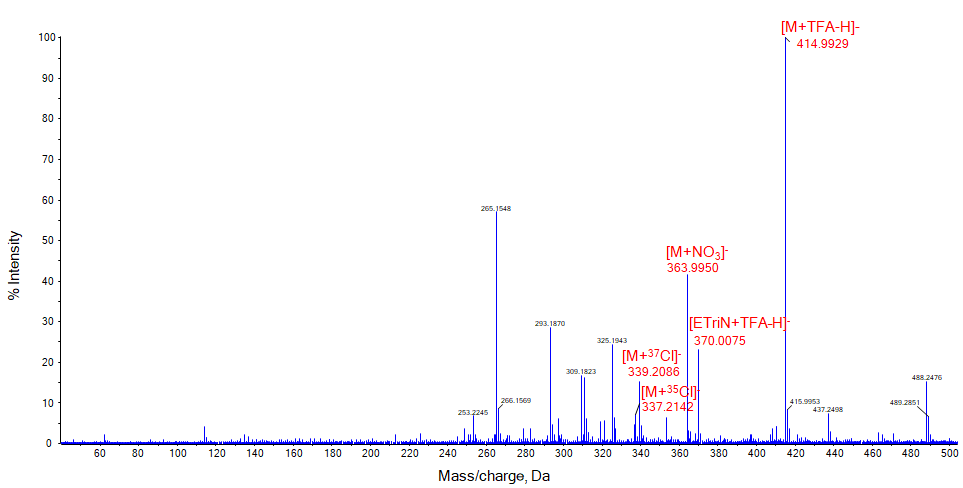


Figure S4 SAWN-MS spectrum of 1 ng/mL ETN in MeOH, analyzed in negative ionization mode. Peaks labeled in red were identified as adducts of ETN.


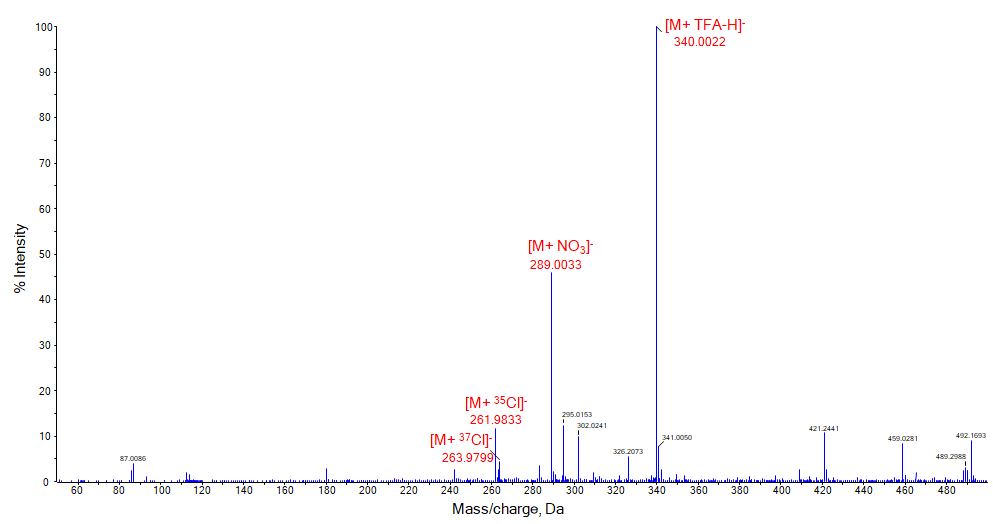


Figure S5 SAWN-MS spectrum of 1 μg/mL NG in MeOH, analyzed in negative ionization mode. Peaks labeled in red were identified as adducts of NG.


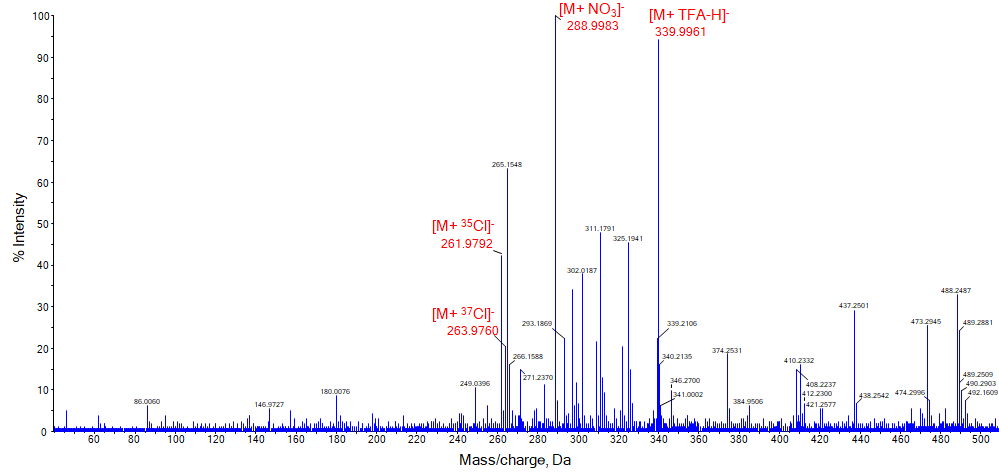


Figure S6 SAWN-MS spectrum of 10 ng/mL NG in MeOH, analyzed in negative ionization mode. Peaks labeled in red were identified as adducts of NG.


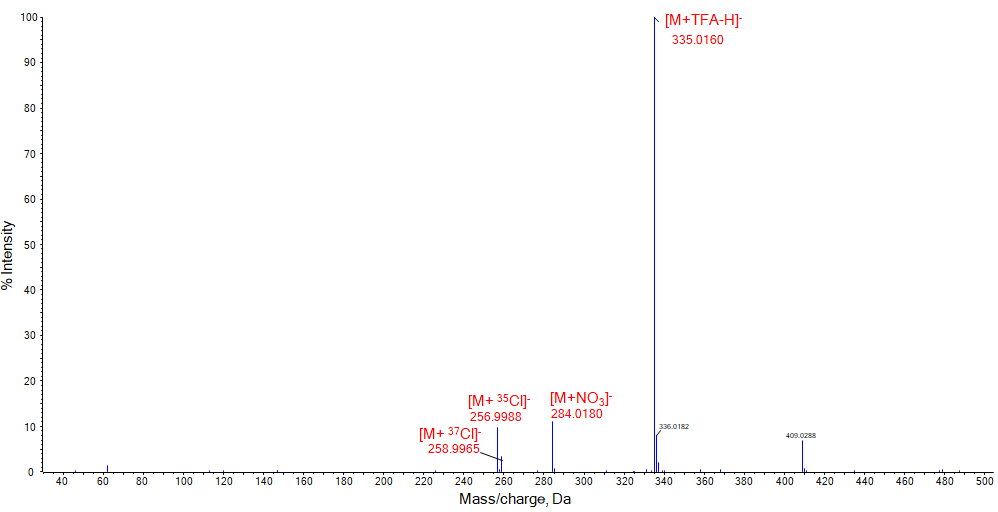


Figure S7 SAWN-MS spectrum of 1 μg/mL RDX in MeOH, analyzed in negative ionization mode. Peaks labeled in red were identified as adducts of RDX.


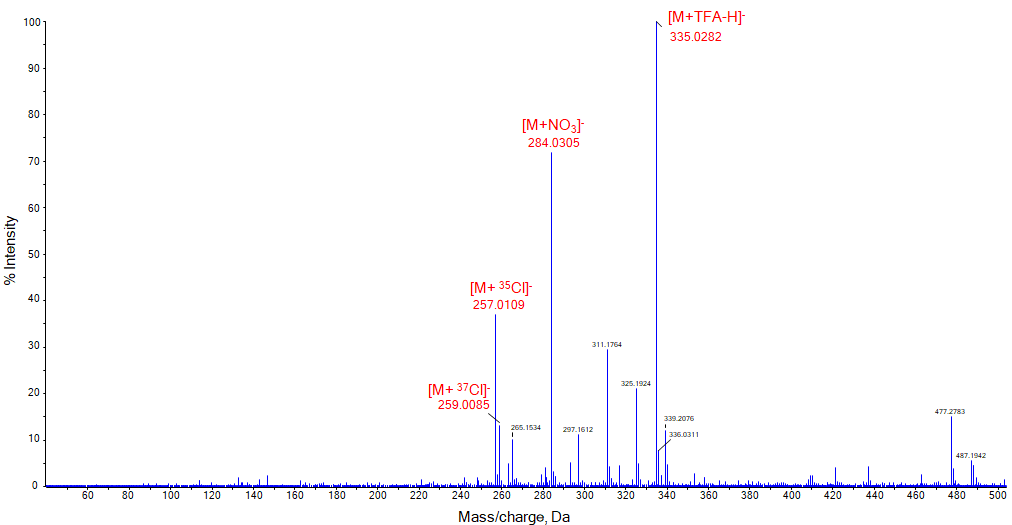


Figure S8 SAWN-MS spectrum of 1 ng/mL RDX in MeOH, analyzed in negative ionization mode. Peaks labeled in red were identified as adducts of RDX.


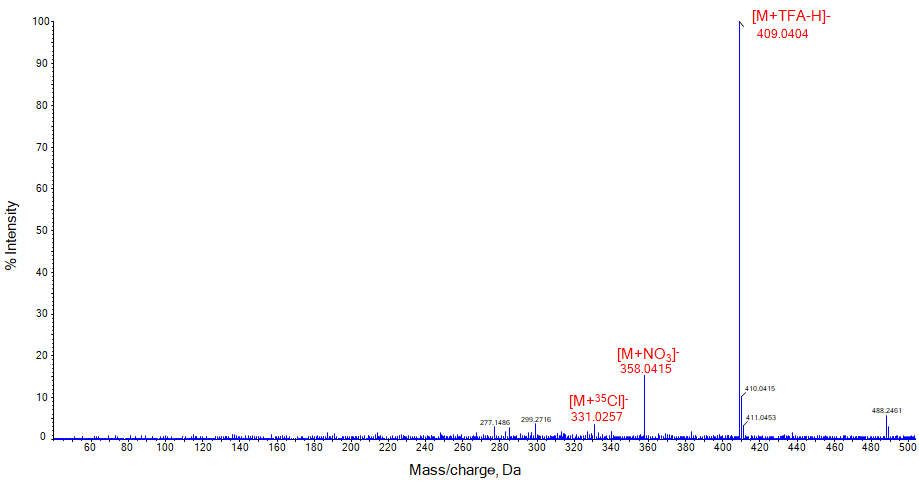


Figure S9 SAWN-MS spectrum of 1 ng/mL HMX in MeOH, analyzed in negative ionization mode. Peaks labeled in red were identified as adducts of HMX.


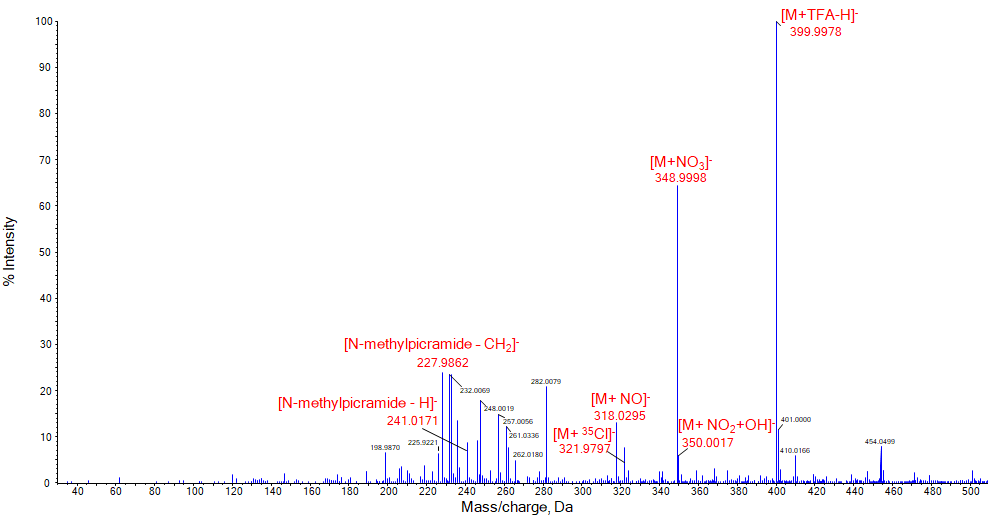


**Figure S10** SAWN-MS spectrum of 1 μg /mL Tetryl in MeOH/H_2_O (70:30 v/v%) analyzed in negative ionization mode. Peaks labeled in red were identified as adducts of Tetryl.

**
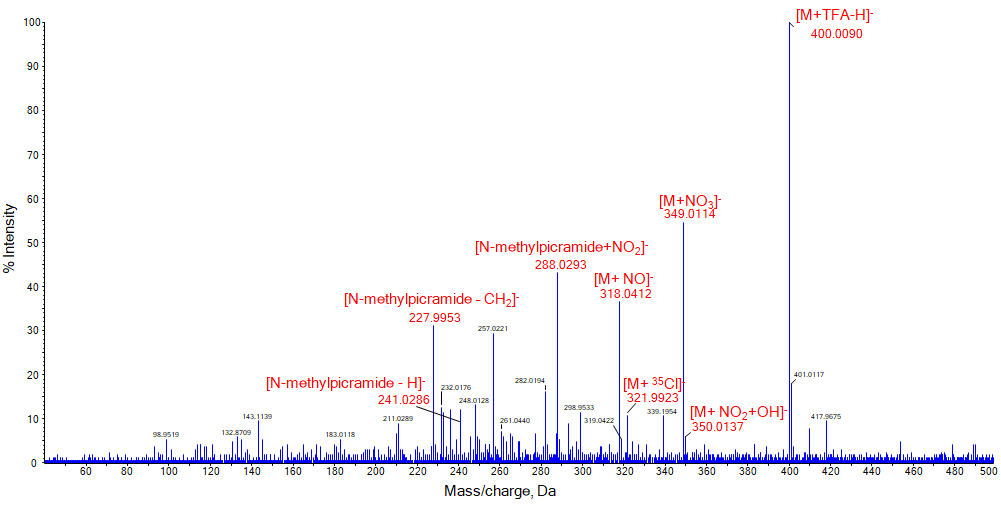
**

**Figure S11** SAWN-MS spectrum of 10 ng/mL Tetryl in MeOH/H_2_O (70:30 v/v%) analyzed in negative ionization mode. Peaks labeled in red were identified as adducts of Tetryl.

**
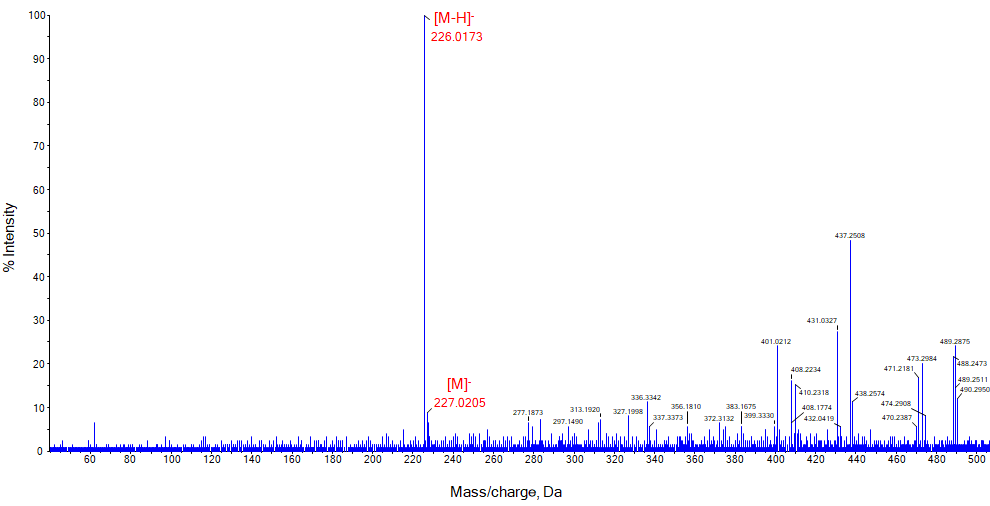
**

**Figure S12** SAWN-MS spectrum of 10 ng/mL TNT in MeOH, analyzed in negative ionization mode. Peaks labeled in red were identified as TNT related ions.


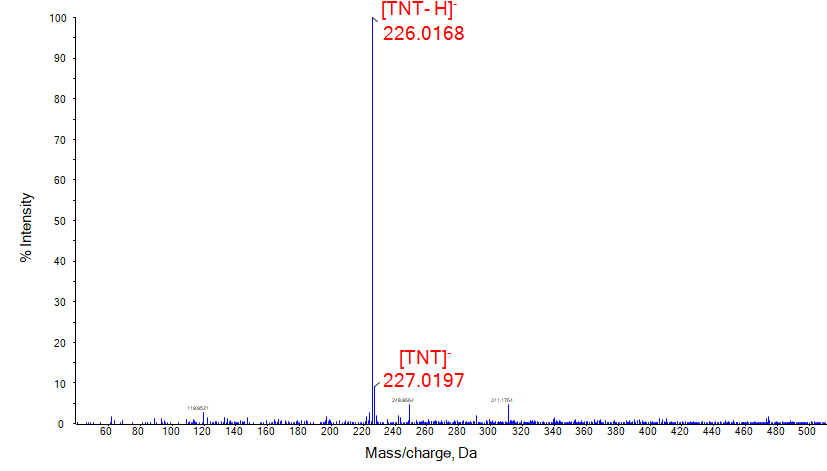


Figure S13 SAWN-MS spectrum of anonymous case sample extract 5 (pre-explosion) in MeOH showing the presence of TNT.


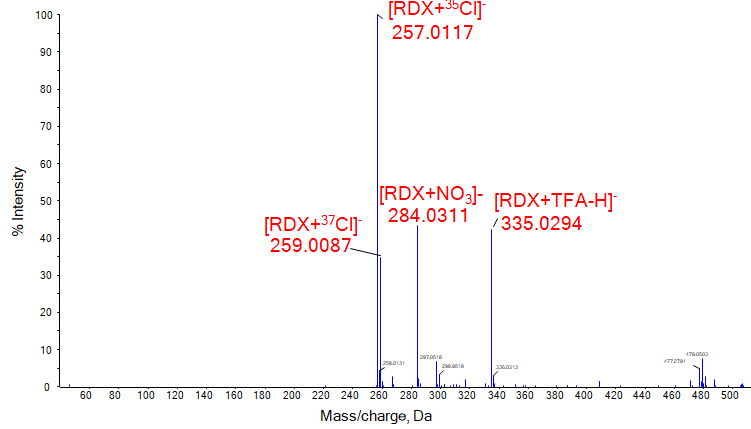


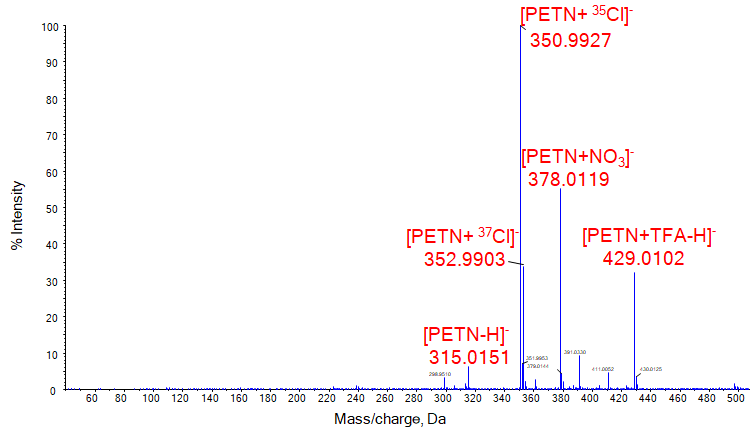
Figure S14 SAWN-MS spectrum of anonymous case sample extract 6 (pre-explosion) in MeOH showing the presence of RDX.

Figure S15 SAWN-MS spectrum of anonymous case sample extract 7 (pre-explosion) in MeOH showing the presence of PETN.


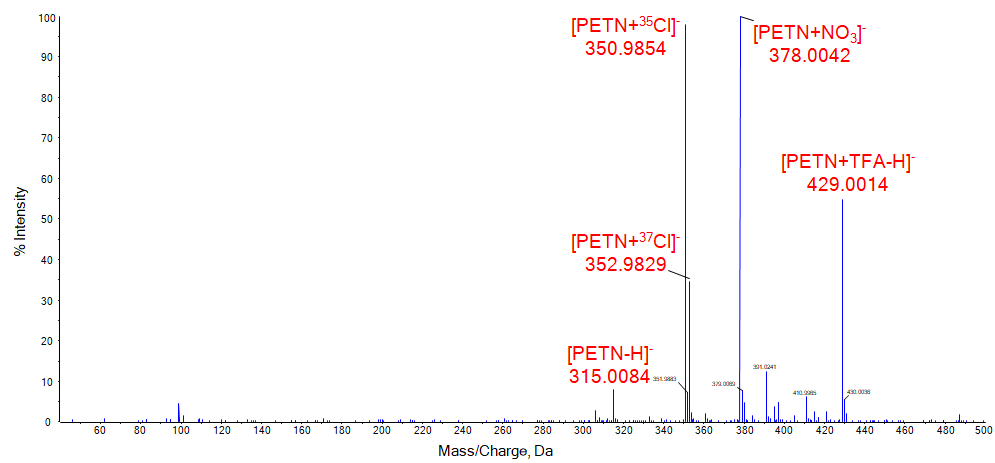


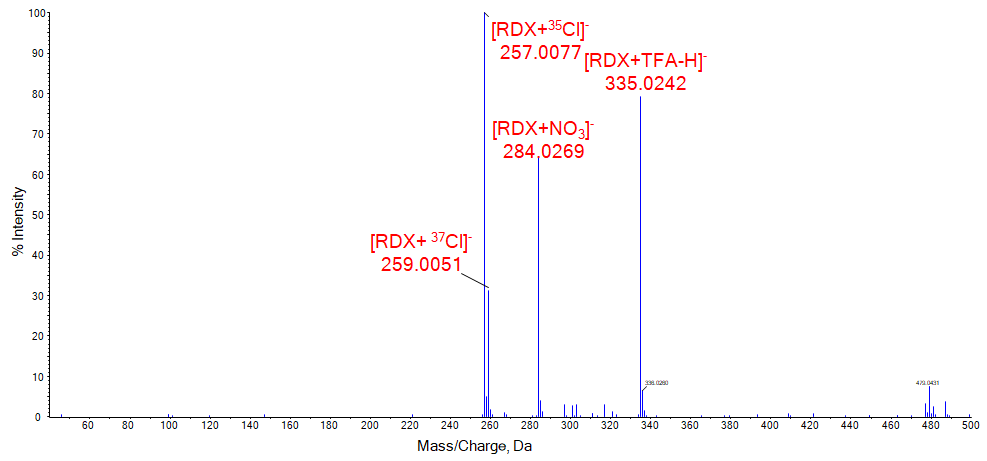
Figure S16 SAWN-MS spectrum of anonymous case sample extract 12 (pre-explosion) in MeOH showing the presence of PETN.


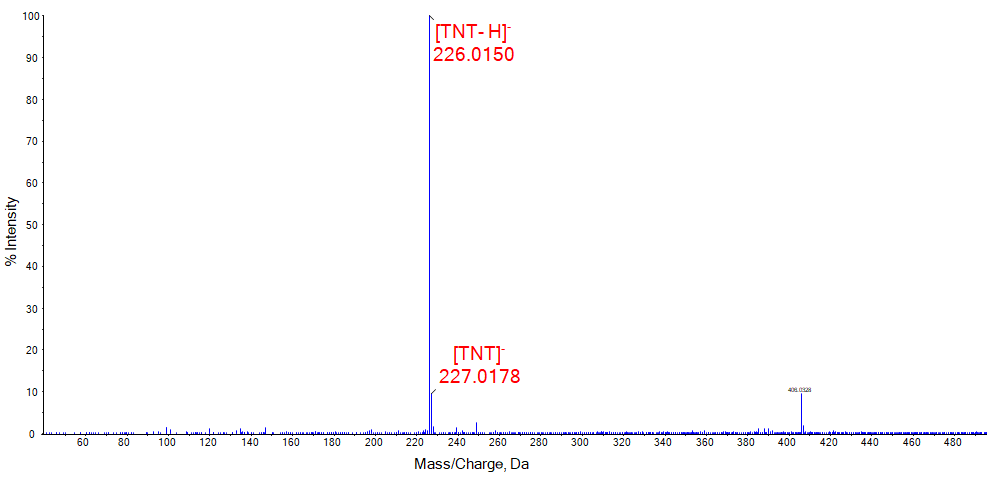
Figure S17 SAWN-MS spectrum of anonymous case sample extract 13 (pre-explosion) in MeOH showing the presence of RDX.

Figure S18 SAWN-MS spectrum of anonymous case sample extract 14 (pre-explosion) in MeOH showing the presence of TNT.


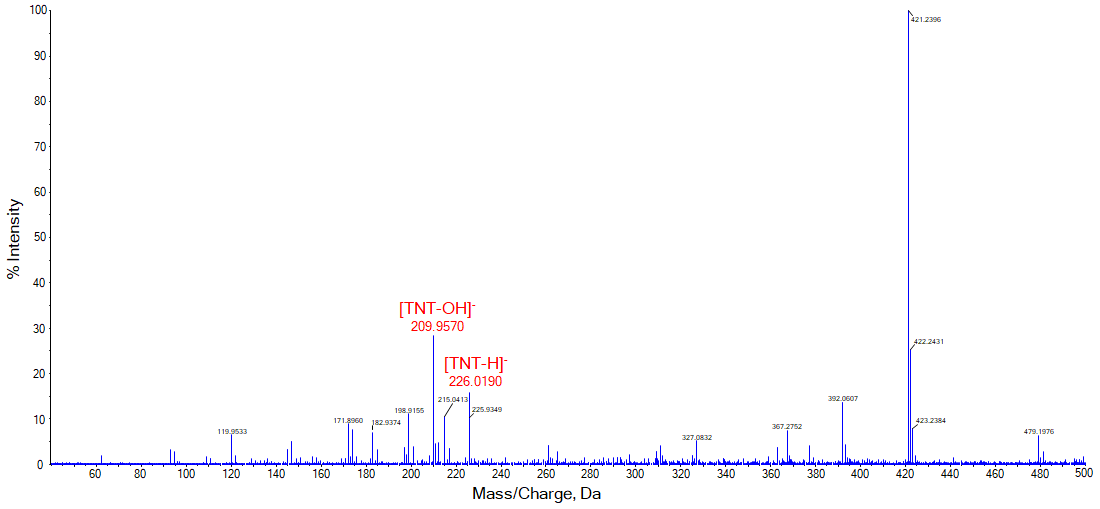


Figure S19 SAWN-MS spectrum of anonymous case sample extract 8 (post-explosion) in MeOH + 1 v% CHCl3 showing the presence of TNT.


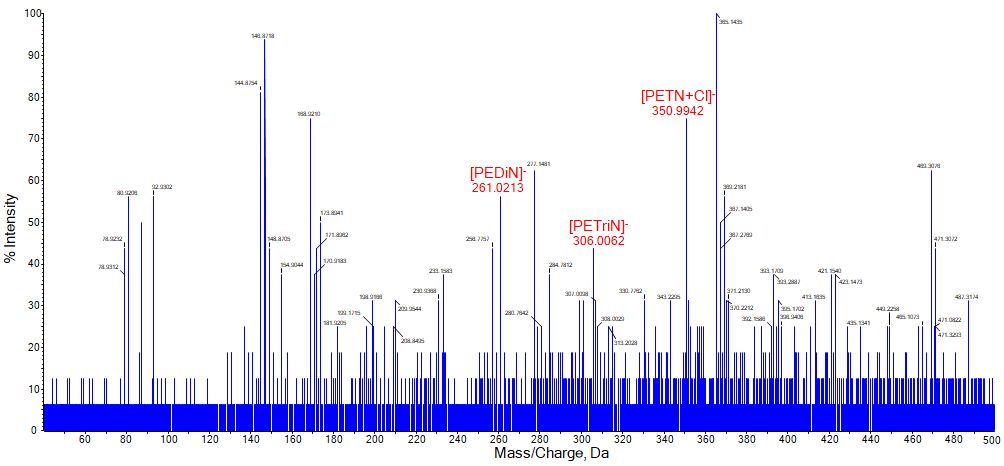


Figure S20 SAWN-MS spectrum of anonymous case sample extract 9 (post-explosion) in MeOH + 2 v% CHCl3 showing the presence of PETN.
